# Genome targeting by hybrid Flp-TAL recombinases

**DOI:** 10.1038/s41598-020-74474-2

**Published:** 2020-10-15

**Authors:** Eugenia Voziyanova, Feng Li, Riddhi Shah, Yuri Voziyanov

**Affiliations:** 1 School of Biological Sciences, Louisiana Tech University, 1 Adams Blvd., Ruston, LA 71272 USA; 2 Present Address: Department of Medicine, Columbia University Medical Center, New York, NY 10032 USA

**Keywords:** Molecular biology, DNA recombination, Systems biology, Genomic engineering, Biological techniques, Genetic engineering, Biotechnology, Gene therapy, Targeted gene repair

## Abstract

Genome engineering is a rapidly evolving field that benefits from the availability of different tools that can be used to perform genome manipulation tasks. We describe here the development of the Flp-TAL recombinases that can target genomic *FRT*-like sequences in their native chromosomal locations. Flp-TAL recombinases are hybrid enzymes that are composed of two functional modules: a variant of site-specific tyrosine recombinase Flp, which can have either narrow or broad target specificity, and the DNA-binding domain of the transcription activator-like effector, TAL. In Flp-TAL, the TAL module is responsible for delivering and stabilizing the Flp module onto the desired genomic *FRT*-like sequence where the Flp module mediates recombination. We demonstrate the functionality of the Flp-TAL recombinases by performing integration and deletion experiments in human HEK-293 cells. In the integration experiments we targeted a vector to three genomic *FRT*-like sequences located in the β-globin locus. In the deletion experiments we excised ~ 15 kilobases of DNA that contained a fragment of the integrated vector sequence and the neighboring genome sequence. On average, the efficiency of the integration and deletion reactions was about 0.1% and 20%, respectively.

## Introduction

The relative simplicity of modifying the target specificity of site-specific nucleases, particularly in the CRISPR/Cas9 and TALEN systems, made these DNA manipulation enzymes the leaders of the genome engineering field^1,2^. However, the characteristic properties of the nuclease systems, such as the necessity to introduce double strand breaks and the reliance on the cell DNA repair machinery to process these breaks, increase the probability of abnormal genome rearrangements and limit the usage of these systems primarily to proliferating cells. To overcome these limitations, researchers devise alternative genome engineering approaches that utilize different DNA manipulation mechanisms. Some of these approaches exploit catalytically inactive dCas9 or Cas9 nickase that can be fused with deaminases^3,4^ or reverse transcriptase^5^, respectively. Other approaches take advantage of the unique DNA manipulation mechanisms of the tyrosine or serine site-specific DNA recombinases^6–13^.

The tyrosine recombinases, such as popular genome engineering tools Flp and Cre, are highly specific for their targets, versatile in performing DNA manipulation reactions, can be easily regulated, and are self-sufficient as they do not require cellular enzymes to complete the reactions. These features made Flp and Cre the systems of choice for creating model cells and organisms in which genome fragments can be efficiently deleted or replaced^14–16^. However, since applications of Flp and Cre require that the target sequences for these recombinases are pre-introduced into a genome locale of interest, these wild-type recombinases cannot be directly used to manipulate the genome of unmodified cells. On the other hand, it was shown that Flp and Cre can be modified to generate recombinase variants that are able to recognize genome sequences that resemble native recombination targets, target-like sequences^8,9,11,17^. This research opens the opportunity to use all the applications that were developed for wild-type Flp and Cre but in original, patient-specific cells.

The number of the target-like sequences in mammalian genomes is quite substantial: the human genome, for example, contains about 600,000 sequences that have different level of homology to *FRT*, the native recombination target for the Flp recombinase^17,18^. This number of the *FRT*-like sequences corresponds to one sequence per ~ 5 kb; such density of these target-like sequences allows DNA manipulations in essentially all genome locales.

It was shown that tyrosine recombinases lack well-defined DNA binding motifs with clear rules that specify which residues have to be mutated to achieve the desired target specificity^19,20^. Furthermore, the extensive mutational analysis of Flp and Cre suggests that the entire tyrosine recombinase molecule participates in the functional target recognition^9,11^. Consequently, the main approach that is used to generate target-specific variants of tyrosine recombinases is random target-linked mutagenesis, although the modification of the residues that directly contact DNA can speed up the protein evolution process^8,9,11,17,21,22^.

A different approach was used to engineer target-specific variants of serine recombinases that are composed of two domains with distinct function: target binding and catalytic^6^. In this approach, a modified catalytic domain with relaxed target specificity is fused with the DNA binding domain, target specificity of which can be easily programmed. The approach was successfully employed to create zinc-finger recombinases, or ZFRs, TALE recombinases, or TALERs, and Cas9 recombinases, or recCas9, that were created by fusing the activated catalytic domains of the invertase Gin or the resolvase Tn3 with the DNA binding domains of either zinc fingers, TAL effectors, or the catalytically inactive Cas9 protein, respectively^7,10,12,13^.

This modular protein design concept, in which proteins with different functional properties are fused together, was also successfully used to develop hybrid site-specific nucleases: zinc finger nucleases, or ZFNs, and transcription activator-like effector nucleases, or TALENs, that are composed of a nonspecific DNA nuclease FokI and the respective DNA binding domains with programmable target specificity^23,24^. Target affinity and specificity in these modular systems can be modified by changing the number of the target recognizing units in their DNA binding domains to achieve the optimal balance between target specificity and non-specific DNA binding^1,25–30^.

In principle, the modular protein design can be applied to generate hybrid tyrosine recombinases, in which a recombinase variant could be stabilized on their targets by an extra target-specific DNA binding domain. Theoretically, two types of hybrid tyrosine recombinases can be engineered. In the first type, both the recombinase and the extra DNA binding modules can have unique specificity for a genomic target-like sequence of interest. In the second type, the extra DNA binding module can be specific for a particular target-like sequence while the recombinase module can have specificity for several, if not the majority of the target-like sequences. Because of the functional differences of their recombinase modules, the two types of the hybrid recombinase should differ in the overall target specificity (higher for the first type and lower for the second type) and in the amount of technical effort needed to engineer these hybrid enzymes (again, higher for the first type and lower for the second type). Despite the differences in the overall target specificity, both types are expected to recombine just the target sequence of interest. The broad target specificity of the recombinase module in the second type of the hybrid recombinases should simplify the modification of its specificity: the process will be reduced just to the replacement of the extra DNA-binding module by the respectively reprogrammed one.

In this proof-of-principle work we analyzed the suitability of both types of the hybrid tyrosine recombinases for genome manipulations. For this, we engineered Flp-TAL recombinases which are composed of a Flp recombinase variant with either narrow or broad specificity for the *FRT*-like sequences and the DNA binding domain of the TAL effector, TAL DBD. We examined the integration and deletion activities of the Flp-TAL recombinases in intact human HEK-293 cells and found that only Flp-TAL but not their respective ‘plain’ target-specific Flp variants were able to efficiently perform these reactions. We also demonstrated that the Flp variant with broad specificity can be fused with the TAL modules of different specificity to direct the recombinase to target the desired genomic *FRT*-like sequences. On average, the efficiency of the integration and deletion reactions mediated by the Flp-TAL recombinases was about 0.1% and 20%, respectively, which is comparable to that of Flp^31–33^. Our results suggest that hybrid tyrosine recombinases can be developed into a promising genome engineering platform.

## Results

### Flp-TAL recombinase architecture

**Figure 1:**
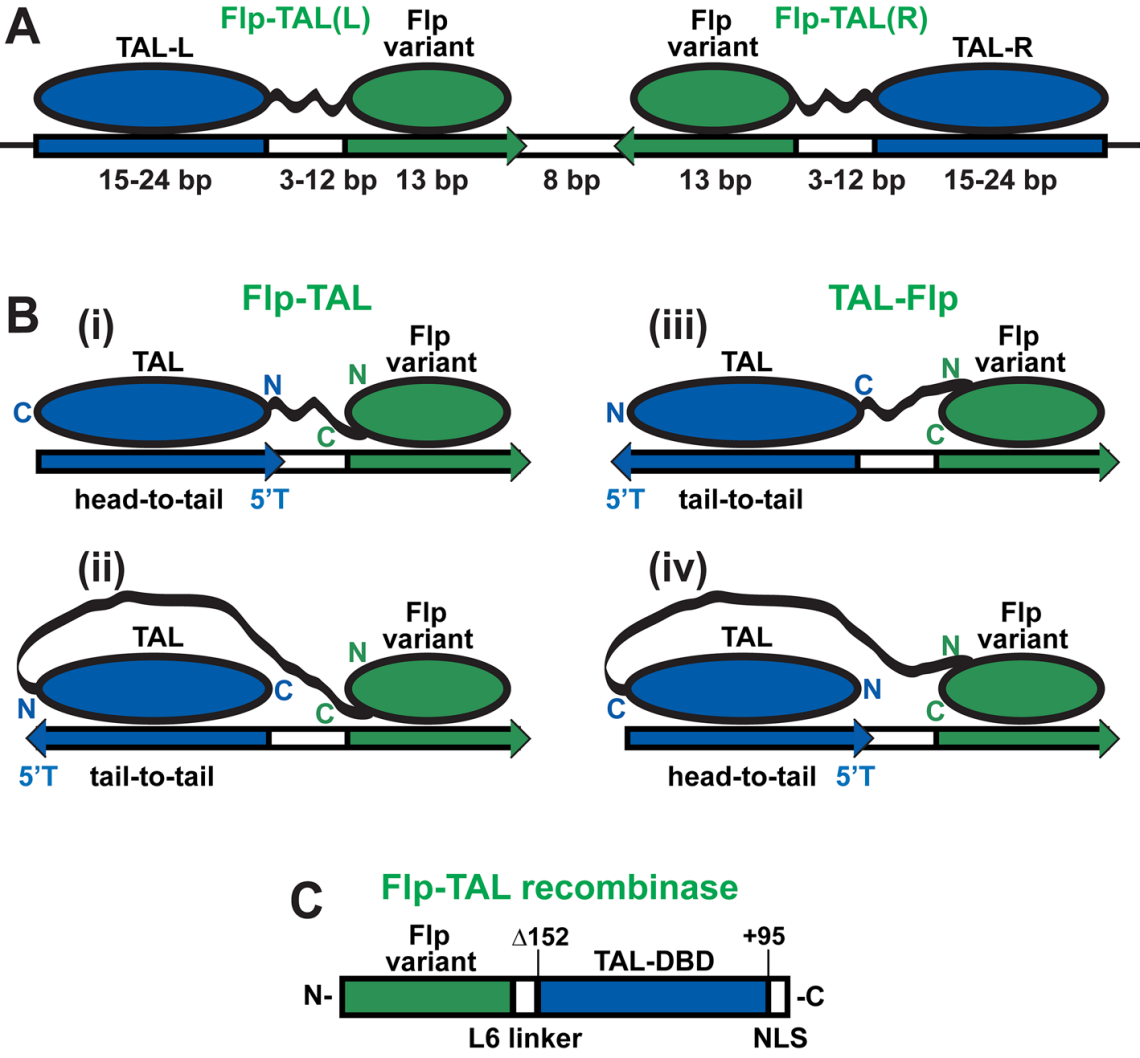
Flp-TAL recombinases. (**A**) General mode of target binding by Flp-TAL recombinases. (**B**) Flp-TAL and TAL-Flp arrangements of the Flp and TAL modules and the respective possible relative arrangements of the Flp and TAL recognition sequences. (**C**) Schematic of the Flp-TAL recombinase.

The mode of the target binding by the Flp-TAL recombinases is specified by the way the catalytic module of the hybrid recombinase, Flp, binds to its native recognition sequence, *F*lp *R*ecombination *T*arget, or *FRT* (Fig. 1A): the Flp module binds to the inner segments of the hybrid target sequence (that is, to the *FRT*-like sequence), while the TAL module—to the outer segments. The analysis of the Flp/DNA and the TAL DBD/DNA complexes^20,34^ shows that, in principle, hybrid Flp-TAL recombinases can be engineered by fusing the TAL module either to the C-terminus or to the N-terminus of the Flp module thus generating enzymes with the Flp-TAL or TAL-Flp architectures, respectively [Fig. 1B (i, ii) and (iii, iv)].

Both the Flp-TAL and TAL-Flp recombinases can be engineered to bind to two possible arrangements of the inner and outer segments of the target sequences: head-to-tail and tail-to-tail (Fig. 1B). Depending on the arrangement, the hybrid recombinases can be engineered either with a short linker that connects the Flp and TAL modules [thus generating the compact hybrid enzyme architecture, Fig. 1B (i) and (iii)] or with a long linker [thus generating the stretched enzyme architecture, Fig. 1B (ii) and (iv)]. Functionally, the compact architecture is preferred since the shorter linker is likely to elicit stronger target stabilizing effect of the TAL module on the Flp module.

To pilot test the hybrid tyrosine recombinase system, we decided to proceed with the compact Flp-TAL architecture and the corresponding head-to-tail arrangement of the target sequences, Fig. 1B (i). Our decision was influenced by the following considerations. (1) Flp-TAL, in contrast to TAL-Flp, is a C-terminal Flp fusion and such fusions usually do not lower the activity of Flp (our unpublished observations) and therefore Flp-TAL is expected to be more active than TAL-Flp. (2) The head-to-tail target sequence arrangement has an important advantageous functional property: it allows testing TAL-binding sequences of different length but the same 5′T nucleotide without the need to respectively adjust the length of the linker between the Flp and TAL modules to keep the interface between the Flp and TAL modules the same [compare Fig. 1B (i) and (ii)].

In the Flp-TAL design we utilized the TAL DNA-binding domain and the inter-modular linker that were proven in other hybrid enzymes (Fig. 1C and Supplementary Fig. 1). In this design, the full-length Flp recombinase variant is fused with the TAL DNA-binding domain core that starts at the position + 152 (Δ152 truncation of the N-terminal segment of the TAL effector) and ends at the position + 95 of the C-terminal segment of the TAL effector^1^. The Flp and TAL modules are connected via linker L6 that was shown to support the highest level of activity of the zinc-finger recombinases (Supplementary Fig. 1 and Ref.^10^).

### Rationale for generating Flp-TAL recombinases with Flp modules of different target specificity

In order to target Flp-TAL recombinases to genomic *FRT*-like sequences of interest, target specificity of both Flp and TAL modules has to be respectively adapted. While the modification of the target specificity of the TAL module is a simple programming and assembly task^28^, the alteration of the target specificity of the Flp module is not as straightforward and requires several (and quite often multiple) rounds of protein evolution^9,17,35,36^. The active use of Flp-TAL recombinases in genome engineering applications can be facilitated if a simple approach to evolve Flp modules is developed.

Previous research on altering target specificity of the Flp recombinase showed that the first several rounds of the protein evolution experiments usually yield two types of Flp variants^9,37^: those that preferentially recombine the desired *FRT*-like sequence and those that can recombine several *FRT*-like sequences with about the same efficiency. In principle, both types of Flp variants can be used as modules for Flp-TAL recombinases. The hybrid enzymes with the highly specific Flp modules are expected to have exceptional target specificity and recognize just the *FRT*-like sequences of interest. On the other hand, the modification of the target specificity of these Flp-TAL recombinases is expected be laborious since a new Flp variant has to be evolved for a particular *FRT*-like sequence.

Flp-TAL recombinases with the Flp modules that are able to recombine many, if not the majority of the high-scoring *FRT*-like sequences^17^ are expected to have lower specificity. Nevertheless, as explained in Supplementary Note 1, target specificity of these hybrid recombinases should be sufficiently high to target just the *FRT*-like sequences of interest. The main advantage of these Flp-TAL recombinases will be the ease of their target modification: what will be required is just the replacement of the TAL module with that reprogrammed to target the sequence of interest.

### Genomic *FRT*-like sequences

**Figure 2:**
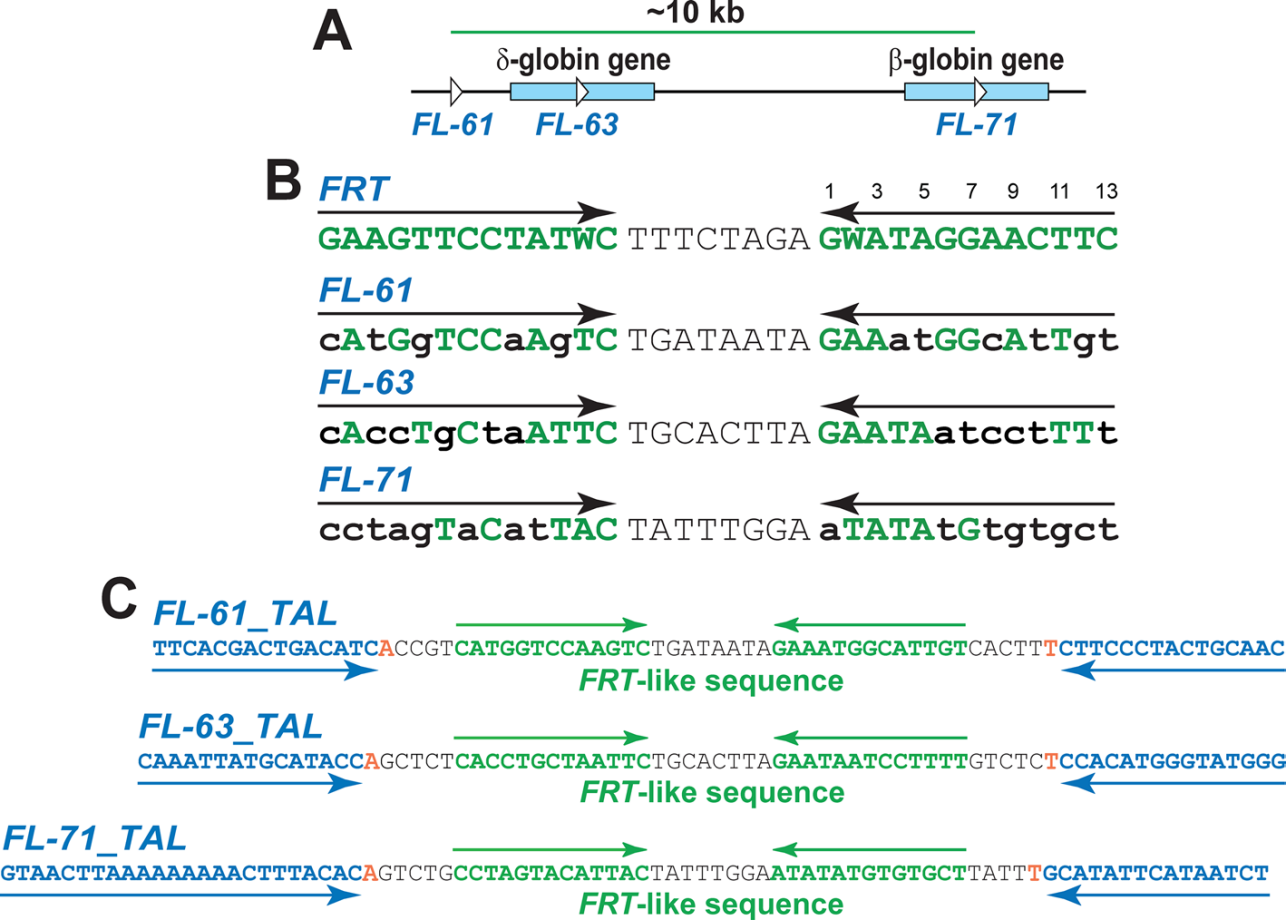
*FRT*-like sequences *FL-61*, *FL-63*, and *FL-71*. (**A**) Relative location of the *FL-61*, *FL-63*, and *FL-71* sequences in the human genome. (**B**) Alignment of *FRT*, *FL-61*, *FL-63*, and *FL-71*. Base pairs in *FL-61*, *FL-63*, and *FL-71* that differ from the corresponding base pairs in *FRT* are shown as lower case bold black letters. (**C**) *FL-61_TAL*, *FL-63_TAL*, and *FL-71_TAL* sequences. 5′T bases for the TAL recognition sequences (marked by blue arrows) are shown in red.

In this work we pilot-tested the Flp-TAL system on three *FRT*-like sequences that are located in the human β-globin locus: upstream of the δ-globin gene and in the δ-globin and β-globin genes: *FL-61*, *FL-63*, and *FL-71*, respectively, which are separated from each other by 2.7 kb and ~ 7.5 kb, respectively (Fig. 2A,B).

Genomic *FRT*-like sequences can have several potential upstream and downstream TAL recognition sequences since the requirements for the TAL recognition sequences are quite relaxed: the only major prerequisite is a thymine at the position N-1 of the sequence, 5′T^38^. We hypothesized that the TAL modules have to recognize DNA sequence of 15–24 bp in length to be useful in targeting Flp-TAL recombinases to the desired *FRT*-like sequence and to be easily manageable from the technical point of view. We also hypothesized that the TAL binding sequence should be separated from the recombinase binding element of the *FRT*-like sequence by 3 to 12 base pairs to avoid steric clashes between the Flp and TAL modules or their significant spatial separation that might impair functional performance of the Flp-TAL recombinases.

Additionally, we hypothesized that the length of the TAL binding sequences should correlate with the degree of similarity between the Flp binding elements of the *FRT*-like sequence and that of *FRT*: the weaker the similarity, the longer the TAL binding sequence.

Based on the above hypotheses, we wanted to test the functionality of the 15-bp long TAL binding sequences (except for the upstream 24-bp long TAL binding sequence for *FL-71*) that are separated from *FL-61*, *FL-63*, and *FL-71* by 4–5 bp (Fig. 2C).

### Evolution of Flp variants capable of recombining *FL-61*, *FL-63*, and *FL-71*

As a genome engineering tool, the Flp-TAL system would benefit if its Flp modules can be evolved quickly. We therefore tested whether these modules can be generated in just one round of protein evolution. Such task necessitates that the process starts not from wild-type Flp but from a library of Flp variants that already contain some of the mutations that are needed to acquire the desired target specificity. Over the course of our research we evolved a number of Flp variants that recognize different genomic targets^9,17,22^. In addition to unique mutations, these enzymes contain a group of mutations that is frequently present in all variants. Most likely, these common mutations collectively relax the strict target specificity of Flp and allow it to recombine not only *FRT* but also *FRT*-like sequences. The unique mutations in these Flp variants can either further relax or, in contrast, narrow the variant’s target specificity.

Some of the Flp variants that bear the common as well the unique mutations can be used as templates to speed up the evolution by shuffling their genes to generate libraries which can be then screened to identify the variants with the desired target specificity. The pool of the template genes can be enriched by including the Flp genes that are randomized at codons 55, 58 and 59 since amino acids at these positions mediate contacts with the first four base pairs of the Flp binding elements of *FRT* that were shown to be the most critical for the Flp-*FRT* recognition^17^.

**Figure 3:**
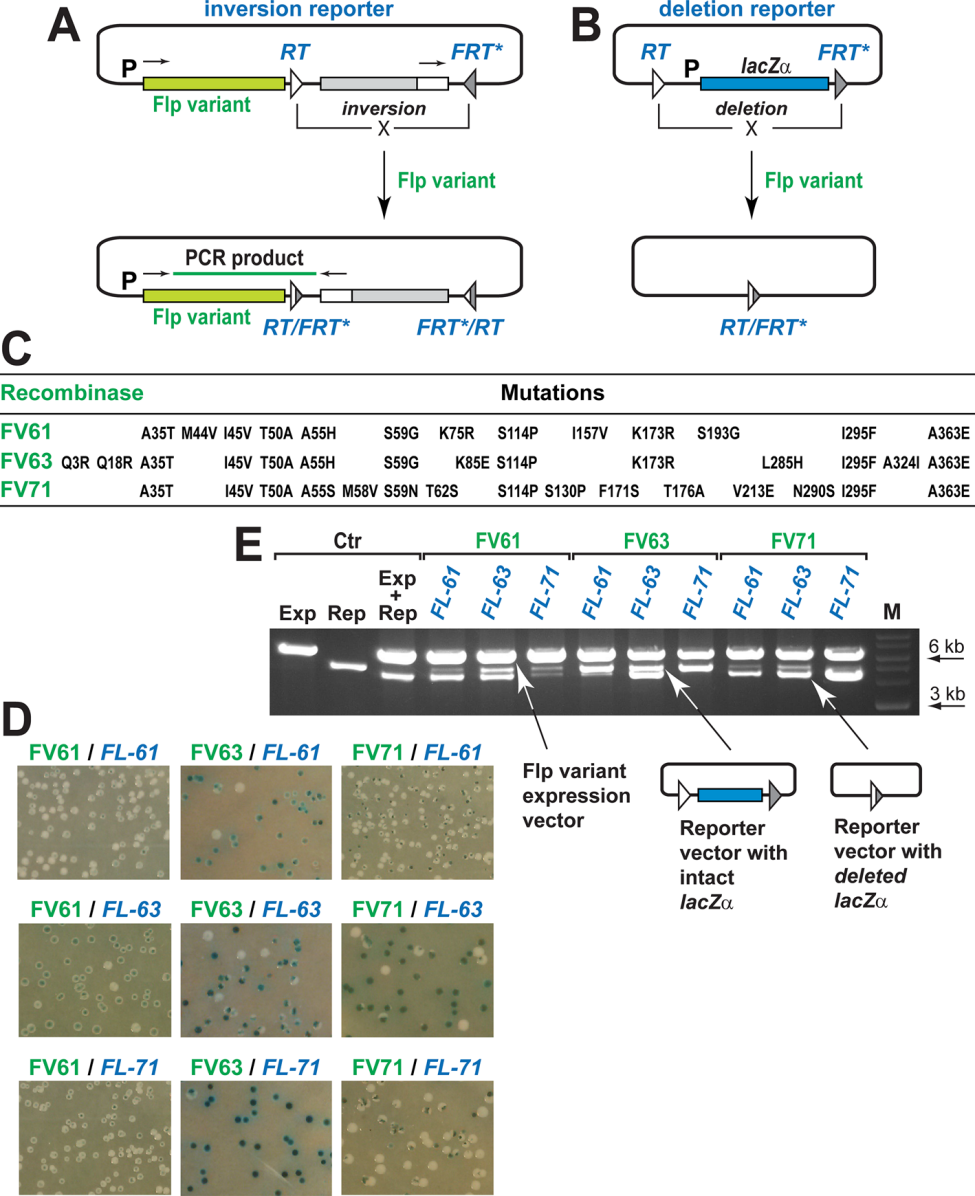
Evolution of Flp-TAL recombinases. (**A**) The inversion assay. The reporter bears the inversion cassette flanked by the recombination targets in the head-to-head orientation: *FL-61*, *FL-63*, or *FL-71* (marked as *RT*) and FRT*, which has the spacer either from *FL-61*, *FL-63*, or *FL-71*, respectively. Upon the expression of a recombination-competent Flp variant, the cassette is inverted so the gene that encodes this variant can be PCR amplified (the small black arrows above the Flp variant gene and the inversion cassette indicate the location and orientation of the PCR primers). (**B**) The deletion assay. The deletion reporter bears the *lacZα* cassette flanked by the respective recombination targets in the head-to-tail orientation. If a Flp variant is able to delete the cassette, bacterial cells, in which the deletion event occurs, form white/partially white colonies when plated on the X-gal containing plates. (**C**) Mutations in the FV61, FV63, and FV71 variants. (**D**) Activity of FV61, FV63, and FV71 on the *FL-61*, *FL-63*, and *FL-71* substrates. The assays were performed using the respective deletion reporters. (**E**) Electrophoregram of the plasmid DNA isolated from the colonies that were pooled from the respective plates shown in (**D**); the plasmid DNA was digested with HindIII that uniquely cuts the expression and reporter plasmids. Ctr, control vectors and vector combinations: Exp, Flpe expression vector; Rep, the deletion reporter that bears the *lacZα* cassette flanked by the *FRT* sequences; Exp + Rep, Flpe expression vector and the respective reporter, in which the *lacZα* cassette was completely deleted; M, DNA ladder (NEB, 2-log). The arrows below the electrophoregram point to the location of the respective vectors.

To screen the Flp gene libraries we designed a two-step system that is composed of the inversion and deletion reporters which are used sequentially (Fig. 3A,B). The reporter cassettes in these vectors are flanked by the pair of the recombination sequences that are arranged in the head-to-head and head-to-tail orientations, respectively. The inversion and deletion reporters have different purpose. The inversion reporter is used to identify a large pool of Flp variants that are able to recombine both the *FRT*-like sequence and *FRT*; these variants are selected by amplifying the Flp variant genes in the inversion-positive configuration of the reporter (Fig. 3A). The deletion reporter is then used to screen the inversion-positive library for the Flp variants that are sufficiently active to delete the reporter cassette in at least some of the vector molecules (Fig. 3B and Ref.^35^).

We tested the ability of the two-step screening system to identify Flp variants with the desired specificity using three recombination target pairs: *FL-61* and FRT*, *FL-63* and FRT*, or *FL-71* and FRT* (FRT* has the wild-type *FRT* sequence except for the spacer that is the same as in the respective *FRT*-like sequence). In these pilot experiments our goal was to test the capabilities of the two-step screening system rather than to exhaustively analyze the resultant Flp libraries. The screening system identified several Flp variants that were able to recombine the *FL-61*/FRT*, *FL-63*/FRT*, and *FL-71*/FRT* pairs with reasonable efficiency. The variants that demonstrated the highest activity on their respective recombination pairs were used in the subsequent experiments and named FV61, FV63, and FV71 (Fig. 3C). These variants were tested for their ability to recombine *FL-61*, *FL-63*, and *FL-71* to identify those that have either strict or broad target specificity.

FV61, FV63, and FV71 responded differently when they were challenged with the ‘non-cognate’ *FRT*-like sequences (Fig. 3D,E). FV63 was the most specific and was able to recombine reasonably well only the *FL-63* sequence; some weak recombination activity was seen on *FL-61* but the recombination activity on *FL-71* was barely detectable. FV71 was the least specific and was able to recombine all three genomic *FRT*-like sequences. FV61 was also able to recombine all these sequences but we noticed an abnormality in its phenotype: the pale blue colonies in the experiments with all *FRT*-likes sequences (Fig. 3D) and the faint reporter bands (both intact and recombined) in the *FL-71* experiments (Fig. 3E). This abnormality suggests that FV61 binds to DNA (or just to the *FRT*-like sequences) apparently tighter than do FV63 and FV71, affecting the plasmid copy number and/or the efficiency of the *lacZα* gene expression. Off note, our evolution experiments sometimes generate Flp variants with the FV61-like phenotype.

The mutational profile of FV61, FV63, and FV71 is shown in Fig. 3C. As anticipated, these Flp variants bear a group of common mutations: at positions 35, 45, 50, 114, 295, and 363. In addition, each variant has a set of unique mutations, some of which were seen in other target-specific Flp variants (positions 44, 62, 130, 173, 176, 193, and 324) while the other unique mutations are new (positions 3, 18, 75, 85, 157, 171, 213, and 290). In addition, FV61 and FV63 have the same set of mutations at positions 55, 58, and 59 which is different from that of FV71; the functional significance of this difference requires further analysis. We also noted that the profile of mutations in FV61, FL63, and FV71 is different from that of the Flp variants that were previously evolved to recombine *FL-61*, *FL-63*, and *FL-71*^17^.

### Flp-TAL variants can integrate a reporter into the desired locations in the human genome

To test whether Flp-TAL recombinases are capable of targeting *FRT*-like sequences in their native environment, we fused the FV61, FV63, and FV71 variants with the TAL modules that were programmed to recognize the respective TAL recognition sequences (Figs. 1A, 2C). Since the TAL modules in the Flp-TAL recombinase have to be specific for the upstream and downstream TAL recognition sequences of the *FRT*-like sequence, two Flp-TAL variants for each hybrid recombinase were engineered: Flp-TAL(L) and Flp-TAL(R), respectively, Fig. 1A. For the sake of simplicity, the ‘left’ and the ‘right’ Flp-TAL variants of the corresponding hybrid recombinases were collectively called FV61-TAL, FV63-TAL, and FV71-TAL.

**Figure 4:**
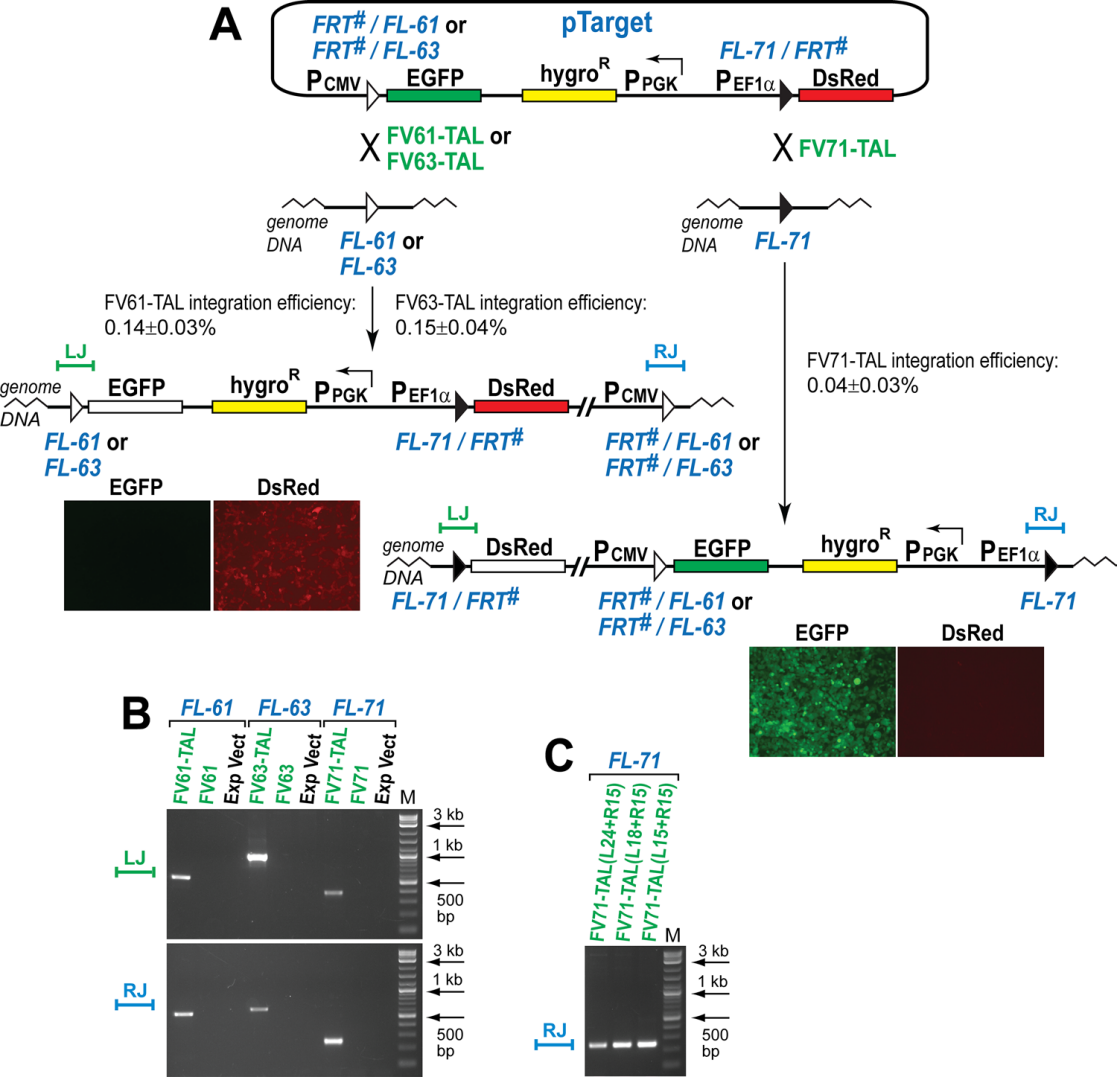
Flp-TAL variants target *FRT*-like sequences *FL-61*, *FL-63*, and *FL-71* in their native chromosomal environment. (**A**) Schematics of the integration assays. The reporter pTarget can be integrated either into *FL-61*, *FL-63*, or *FL-71* depending on the specificity of the Flp-TAL recombinase and the version of pTarget: *FL-61*/*FL-71* or *FL-63*/*FL-71*. Upon integration of pTarget into *FL-61* or *FL-63* the resultant cells become hygromycin resistant and red, while upon integration into *FL-71*, the cells become hygromycin resistant and green (images of the individual expanded hygro^R^/red and hygro^R^/green colonies are shown as examples). The analysis of the individual colonies was performed in two biological replicates. LJ and RJ mark the locations of the left and right junctions of the integrated reporter and genomic DNA, respectively; diagnostic PCR at these locations was used to determine the authenticity of the reporter integration. (**B**) PCR analysis of the pooled hygro^R^ colonies generated in the experiments with the Flp-TAL recombinase, the ‘plain’ recombinase variant, and the ‘empty’ expression vector. LJ, RJ, the PCR analysis of the left and right junctions of pTarget integrated into the respective genomic sequences; M, DNA ladder (NEB, 2-log). (**C**) PCR analysis of the pooled hygro^R^ colonies generated in the integration experiments with three combinations of the FV71-TAL recombinases: FV71-TAL(L24) + FV71-TAL(R15), FV71-TAL(L18) + FV71-TAL(R15) and FV71-TAL(L15) + FV71-TAL(R15). The diagnostic PCR analysis was performed at the right junction.

The targeting activity of the Flp-TAL recombinases was analyzed via the integration and deletion assays (Figs. 4, 6). The schematic of the integration assay is shown in Fig. 4A. The reporter vector pTarget bears three transcriptional units that express EGFP, hygromycin B phosphotransferase (hygro^R^), and DsRed (the puro-2A-DsRed gene was used to express DsRed). In pTarget, the hybrid *FRT*^*#*^/*FL-61* (or *FRT*^#^/*FL-63*) sequence is located between the CMV promoter and the EGFP gene while the hybrid *FL-71/FRT*^#^ sequence is positioned between the EF1α promoter and the DsRed gene (Fig. 4A). In the hybrid recombination sequence, one recombinase binding element and the spacer comes from the respective genomic target sequence while the other recombinase binding element, *FRT*^#^, comes from *FRT* (Supplementary Fig. 2). Two variants of pTarget were generated: with the *FRT*^*#*^/*FL-61*–*FL-71/FRT*^#^ arrangement of the hybrid recombination sequences and with the *FRT*^*#*^/*FL-63*–*FL-71/FRT*^#^ arrangement.

If FV61-TAL (or FV63-TAL) integrates the respective variant of pTarget into the native *FL-61* (or *FL-63*) sequence, the EGFP gene loses its promoter and thus cannot be expressed. The resultant cells should be therefore red and not green (Fig. 4A). If FV71-TAL is capable of integrating pTarget into the native *FL-71* sequence, the DsRed gene loses its promoter and cannot be expressed. The resultant cells should be therefore green and not red (Fig. 4A).

To test the integration activity of the Flp-TAL recombinases, we transfected HEK-293 cells with the vectors that express FV61-TAL, FV63-TAL, or FV71-TAL and the corresponding pTarget variant. 48 h post-transfection, 1/10 of the cells were transferred into medium supplemented with hygromycin and incubated for about 10–14 days until the hygromycin resistant colonies are formed. Four types of colonies were observed: colorless, green and red, just green, and just red. The ratios between these colony types varied significantly between replicates and batches of the hybrid recombinase vectors and HEK-293 cells (Supplementary Table 1). Although the observed ratios depended on the hybrid recombinase used, we did not find an apparent correlation between the colony type ratios and successful integration of the reporter vector into the respective genomic *FRT*-like sequences. We address this phenomenon in the Discussion section.

Once the hygromycin resistant colonies had at least 100–150 cells, they were pooled and their genomic DNA was isolated and subjected to the PCR analysis to assess the specificity of the integration events (Fig. 4B). The analysis revealed that the PCR bands that are expected if pTarget integrated into the desired *FRT*-like sequences can be obtained only in the experiments that were performed with the Flp-TAL recombinases but not in the control experiments with the ‘plain’ Flp variants FV61, FV63, and FV71 or the expression vector (Fig. 4B). The identity of the diagnostic PCR bands was verified by sequencing (Supplementary Fig. 5A–F).

**Figure 5:**
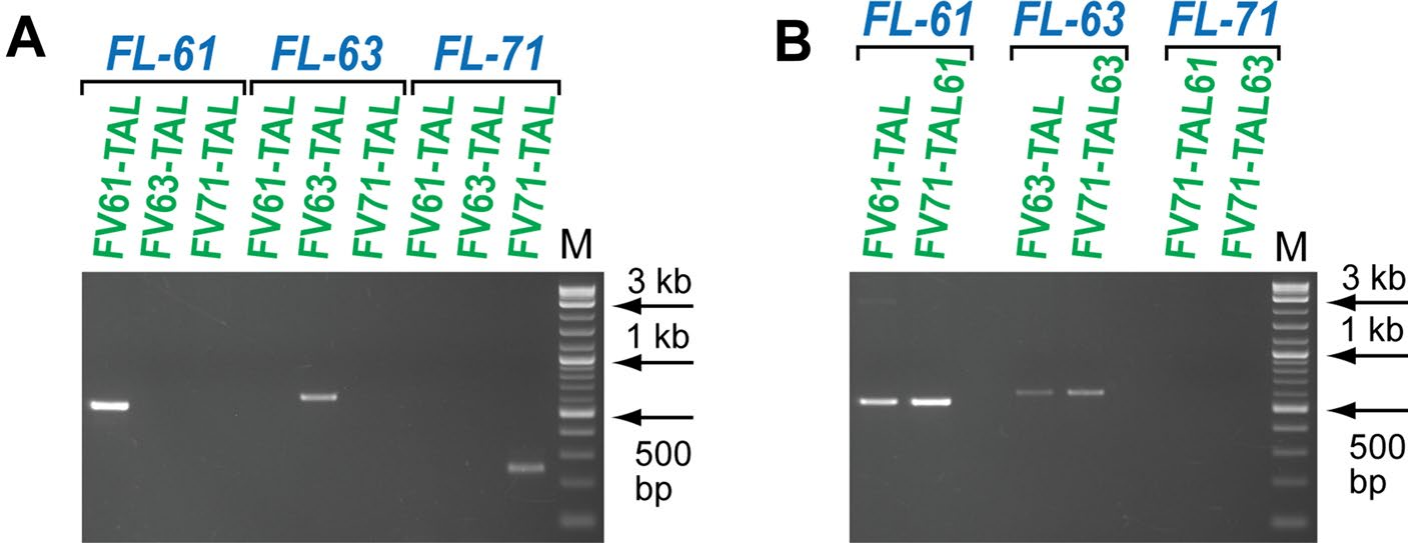
Target specificity of the Flp variants within Flp-TAL is determined by the TAL module. (**A**) pTarget co-transfected with either FV61-TAL, FV63-TAL, or FV71-TAL into HEK-293 cells efficiently targets only the intended *FRT*-like sequence. (**B**) The TAL modules in the Flp-TAL recombinases FV71-TAL61 and FV71-TAL63 variants deliver the FV71 variant to the *FRT*-like sequences *FL-61* and *FL-63* but not to *FL-71*. The diagnostic PCR analyses were performed at the respective right junctions between the integrated pTarget and genomic DNA. M, DNA ladder (NEB, 2-log).

To assess the rate of the Flp-TAL mediated integration, we performed the targeting experiments as described above but instead of pooling all hygromycin resistance colonies we expanded either just red colonies (in the FV61-TAL and FV63-TAL experiments) or just green colonies (in the FV71-TAL experiments) and subjected them to the diagnostic PCR analysis. These experiments showed that the Flp-TAL recombinases integrated the reporter vector, on average, in about 0.1% of transfected cells, although the efficiency of integration into *FL-61* and *FL-63* was about threefold higher than into *FL-71* (Fig. 4A). We also determined that the fraction of the integration-positive colonies was ~ 5–10% of the colonies of the respective color (Supplementary Table 2).

### FV71-TAL targets *FL-71* when both its TAL modules bind to 15-bp sequences

The integration experiments with FV71-TAL were performed with the binary hybrid recombinase that was composed of the ‘left’ variant FV71-TAL(L24) and the ‘right’ variant FV71-TAL(R15), the TAL modules of which bind to the 24-bp long and 15-bp long genomic sequences, respectively. The FV71-TAL(L24) with the longer TAL module was engineered since we hypothesized that the length of the TAL binding sequences should correlate with the degree of similarity between the Flp binding elements of *FRT* and that of the *FRT*-like sequence: the weaker the similarity, the longer the TAL binding sequence. To test the validity of this hypothesis, we engineered two additional FV71-TAL(L) variants: FV71-TAL(L15) and FV71-TAL(L18) with the TAL modules that bind to the 15-bp and 18-bp long sequences, respectively; the binding sequences for these TAL modules start at the same nucleotide as does the sequences for the TAL(L24) module, Fig. 2C. We then tested all three versions of the binary FV71-TAL recombinases [FV71-TAL(L24) + FV71-TAL(R15), FV71-TAL(L18) + FV71-TAL(R15), and FV71-TAL(L15) + FV71-TAL(R15)] for their ability to target *FL-71*. The results of these experiments demonstrated that under the conditions tested the activity of the FV71-TAL recombinase pairs that contained FV71-TAL(L24) was about the same as that of the pairs that contained FV71-TAL(L18) or FV71-TAL(L15), Fig. 4D. Apparently, the high-scoring *FRT*-like sequences^17^, even when one of their recombinase binding elements has relatively low similarity to *FRT*, can be efficiently recombined by the Flp-TAL recombinases, the TAL modules of which bind to the sequences no longer than 15 bp.

### PCR-based assessment of Flp-TAL integration efficiency

We next examined whether PCR analysis can be used to adequately assess the integration efficiency of the Flp-TAL recombinases. For this, we performed PCR analyses of serially diluted genomic DNA isolated from the individual hygromycin-resistance, integration-positive colonies and from the pooled hygromycin-resistance colonies that were generated in the *FL-61* targeting experiments. The lowest concentrations of genome DNA at which we were able to get the integration-specific PCR band were ~ 0.08 ng and ~ 0.63 ng for the individual and for the pooled colonies, respectively (Supplementary Fig. 3A,B).

To translate these results into the number of HEK-293 cells we considered the following: (1) as HEK-293 is an almost triploid cell line^39^, one HEK-293 cell contains about three genome sets and (2) only one out of three chromosomes 11, where the β-globin locus is located, are likely to get modified by the Flp-based recombinases^40^. Consequently, 20 ng of genome DNA, which equals ~ 5500 genomes^7,41^, correspond to ~ 1833 HEK-293 cells. As such, 0.08 ng of genome DNA correspond to ~ 7 HEK-293 cells, while 0.63 ng – to ~ 57 HEK-293 cells. These results suggest that approximately 7 HEK-293 cells can be considered as a practical threshold for determining the lowest number of cells needed to generate the integration-specific PCR band. These results indicated that the proportion of cells in the pooled hygromycin-resistance colonies that were capable of generating the integration-specific PCR band was ~ 1/57. This correlated well with the proportion that was determined based on the number of the integration-positive colonies among the colonies of the respective color: ~ 1/20 to ~ 1/10 (note that the pooled hygromycin-resistance colonies contain not only integration-positive cells but also cells in which the reporter integrated randomly).

We then examined whether the efficiency of the Flp-TAL-mediated integration is sufficiently high to be detected by PCR shortly after transfection, at the cell expansion stage, without the application of selection pressure. Positive answer would indicate that it is realistic to complete the initial testing of various reaction conditions faster, in about 4–5 days, as opposed to about 14–17 days. In these experiments, we tested FV61-TAL and FV71-TAL that were transfected along with pTarget into HEK-293 cells which were expanded 48 h post-transfection and then their genomic DNA was isolated and subjected to the PCR analysis. The analysis of serially diluted genomic DNA showed that the respective integration positive bands can be detected even at this early step when the genomic DNA input was at ~ 10 ng (FV61-TAL experiments) and ~ 40 ng (FV71-TAL experiments), Supplementary Fig. 3C. These inputs correspond to about 2750 and about 11,000 genomes, respectively or about 917 and 3,667 HEK-293 cells (including cells that were not transfected). When the average transfection efficiency in our experiments (~ 10%) is taken into account, these numbers translate into one integration event in ~ 92 of transfected cells (~ 1.08% integration efficiency) in the FV61-TAL experiments and in ~ 367 of transfected cells (~ 0.27% integration efficiency) in the FV71-TAL experiments. Since the integration reaction in Flp recombination is reversible and thus some of the integrated reporter molecules are expected to get deleted, the results of these ‘initial’ integration experiments correlate well with the results of the ‘complete’ integration experiments (Fig. 4A).

### Flp-TAL recombinases are target specific

As the results of the recombination experiments in *E. coli* indicated, two Flp variants: FV71 and FV61, can recombine all three *FRT*-like sequences tested (*FL-61*, *FL-63*, and *FL-71*) while the FV63 variant can recombine mainly *FL-63* (but also *FL-61*, albeit quite weakly), Figs. 3D,E. We next wanted to examine whether the specificity of these Flp variants within Flp-TAL is determined by the TAL module when the hybrid recombinases are assayed in human cells. If a Flp variant retains its original specificity and the TAL module mediates just non-specific stabilization of the Flp module on DNA, without targeting it to the desired *FRT*-like sequence, then Flp-TAL might be able to recombine more than one *FRT*-like sequence and integrate the reporter vector into more than one genomic *FRT*-like sequences. In contrast, if it is the TAL module that determines which particular *FRT*-like sequence is targeted by the Flp-TAL recombinase, then the reporter vector will integrate only into that *FRT*-like sequence.

In these experiments, we co-transfected the Flp-TAL recombinase (either FV61-TAL, FV63-TAL, or FV71-TAL) and the respective pTarget versions (either with the *FL-61*/*FL-71* target sequence pair or with the *FL-63*/*FL-71* target sequence pair, Fig. 4a) into HEK-293 cells and then analyzed which genomic *FRT*-like sequence: *FL-61*, *FL-63*, or *FL-71*, was targeted by the reporter. As the results of these experiments indicated, FV61-TAL, FV63-TAL, and FV71-TAL efficiently targeted only the intended *FRT*-like sequence even when they were challenged with the competing *FRT*-like sequence that was part of the vector sequence (Fig. 5A), confirming the targeting role of the TAL module.

We then wanted to test whether the relaxed specificity variant FV71 can recombine *FL-61* and *FL-63* in human cells when FV71 is fused with the TAL modules that are specific for these *FRT*-like sequences. For this, we engineered the FV71-TAL61 and FV71-TAL63 variants, the TAL modules of which were identical to that in the FV61-TAL and FV63-TAL variants, respectively, but had FV71 as the Flp module. We then performed the integration experiments with FV71-TAL61 and FV71-TAL63 as described previously and PCR analyzed the pooled hygromycin resistant colonies. The results of these experiments demonstrated that FV71-TAL61 and FV71-TAL63 targeted *FL-61* and *FL-63* as efficiently as did FV61-TAL and FV63-TAL (Fig. 5B).

In parallel, we tested if FV61, which also has relaxed specificity, can target *FL-71* if fused to the corresponding TAL modules. Despite extensive experimenting we were unable to detect FV61-TAL71 mediated integration of the reporter vector into *FL-71*. We attributed this failure to an apparent aberrantly tight DNA binding phenotype of FV61 which makes this Flp variant functionally different from FV71.

The results of the ‘TAL module swap’ experiments established an important feature of the Flp-TAL system: if the Flp variant that has broad specificity for the *FRT*-like sequences but lacks abnormally tight DNA binding is used as a module for Flp-TAL, target specificity of such hybrid tyrosine recombinase can be easily modified by respectively reprograming the TAL module.

Next, we examined whether FV71-TAL61, as the hybrid recombinase with the broad target specificity Flp module, integrates the reporter primarily into the *FL-61* sequence (that is specified by the TAL61 module) and not into one of the *FRT*-like sequences with the highest level of homology to *FRT* and the spacer sequence identical to that of *FL-61*. Out of ~ 600,000 *FRT*-like sequences located in the human genome^17^ we identified 29 sequences that meet the above criteria (Supplementary Table 4). The integration experiments were performed as described above and genomic DNA was isolated from the pooled hygromycin resistance colonies. The subsequent PCR analysis did not reveal any apparent integration of the reporter into these *FRT*-like sequences in addition to integrating into *FL-61* (data not shown).

### Flp-TAL recombinases can efficiently delete genome fragments

**Figure 6:**
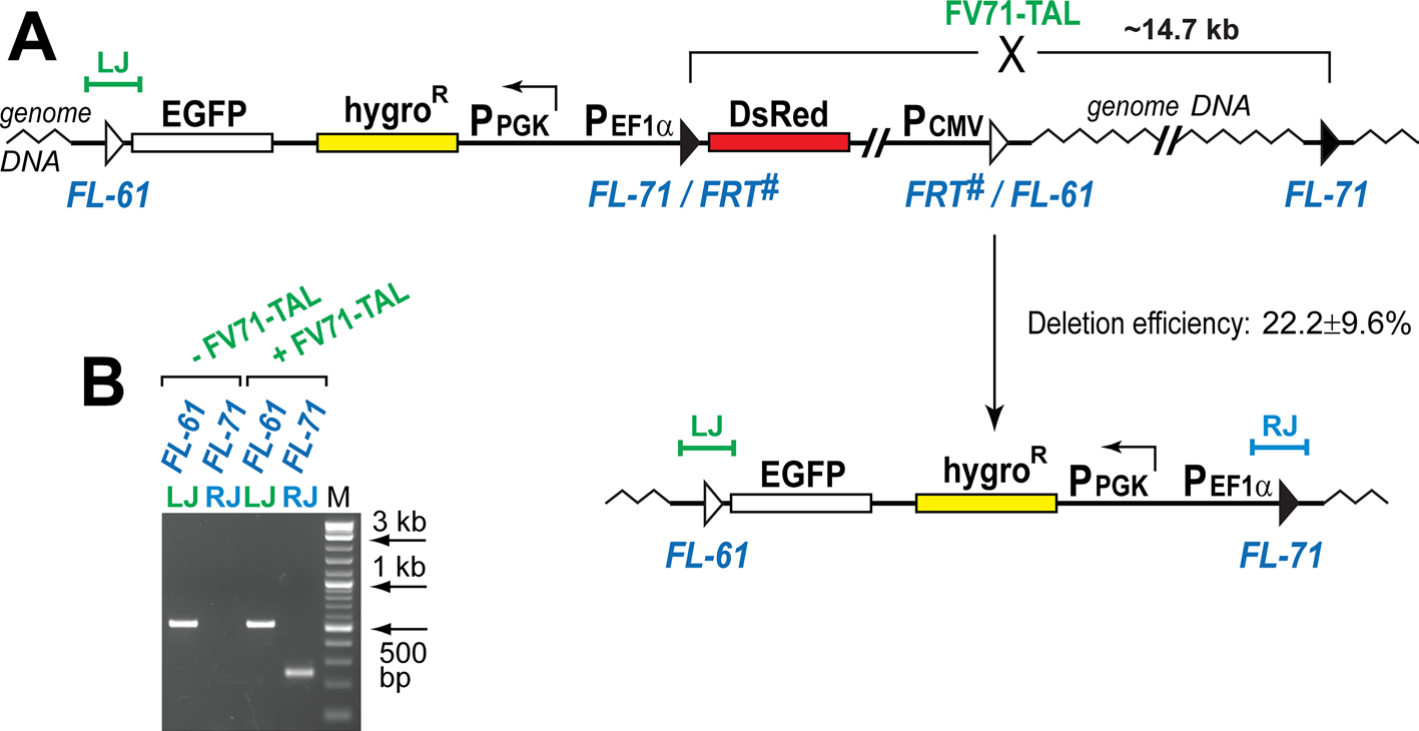
Flp-TAL recombinase can delete large genome DNA fragments. (**A**) Schematic of the deletion assay. Upon expression of FV71-TAL in cells, in which pTarget was pre-integrated into *FL-61* (Fig. 4A), the DNA fragment located between two *FL-71* sequences can be deleted (this DNA fragment contains ~ 4.7 kb of reporter DNA and ~ 10 kb of genome DNA). (**B**) PCR analysis of the FV71-TAL treated cells. The deletion activity was analyzed in three biological replicates (see Supplemental Fig. 4). *FL-61*(LJ) and *FL-71*(RJ), the PCR analysis of the respective left and right vector-genomic junctions of the integrated pTarget before and after the treatment with FV71-TAL. M, DNA ladder (NEB 2-log). (**C**) Sequencing of the deletion-specific PCR product *FL-71*(RJ) confirmed its identity.

Finally, we assessed the ability of the Flp-TAL recombinases to delete large genome fragments. In these experiments we utilized an important functional feature of the pTarget vector that carries two *FRT*-like sequences (Fig. 4A): upon integration of the vector into one of the respective genomic *FRT*-like sequences, the other vector-borne *FRT*-like sequence can be used to delete the DNA fragment that is located between this sequence and the corresponding genomic *FRT*-like sequence (this 14.7 kb DNA fragment contains part of the vector sequence as well as the genomic sequence, Fig. 6A).

In the genome DNA deletion assays we used the integration-positive red cells that were obtained in the FV61-TAL integration experiments (Figs. 4A, 6A). These cells were transfected with the FV71-TAL expressing vectors, expanded, and subjected to the PCR analysis (Fig. 6B), which indicated the appearance of the deletion-specific PCR band, the identity of which was verified by sequencing (Supplementary Fig. 5G). To estimate the number of cells, in which the respective DNA fragment was deleted, we performed the PCR analysis of serially diluted genomic DNA which revealed that the lowest input of genome DNA that was able to generate the deletion specific PCR band was, on average, ~ 0.52 ng (Supplementary Fig. 4) which corresponds to ~ 143 genomes or ~ 48 HEK-293 cells. Since the average transfection efficiency in our experiments was about 10%, these numbers translate into one deletion event in ~ 5 cells or in ~ 20% of transfected cells.

The end result of these deletion experiments was the replacement of the genomic DNA located between *FL-61* and *FL-71* with the respective segment of the pTarget vector (Figs. 4A, 6A). This type of replacement is expected via the dual recombinase-mediated cassette exchange (RMCE) mechanism if both FV61-TAL and FV71-TAL act simultaneously on their respective targets.

## Discussion

In this work we showed that hybrid Flp-TAL recombinases that are composed of the target-specific variants of Flp and the DNA binding domain of the TAL effector, specificity of which can be programmed, are capable of targeting *FRT*-like sequences in their native genome environment. We demonstrated that in Flp-TAL it is the TAL module that determines which *FRT*-like sequence will be targeted.

Two types of Flp-TAL recombinases that differ at the level of target specificity of the Flp modules were engineered: with strict specificity for a unique *FRT*-like sequence and with broad specificity for several *FRT*-like sequences. Since both the Flp and the TAL modules of the former type possess unique target specificity, the hybrid recombinases of this type are expected to be one of the most specific genome editing tools. The obvious limitation of these Flp-TAL recombinases is a substantial effort that is needed to evolve highly specific Flp modules.

In contrast, Flp-TAL recombinases with the Flp module that can target multiple genomic *FRT*-like sequences, offer a simple solution to the problem of modifying their specificity: what is needed is just reprogramming of the TAL module to bind the sequences that flank the *FRT*-like target of interest. Even though the target specificity of these Flp-TAL recombinases is anticipated to be lower than that of the Flp-TAL recombinases with the more specific Flp modules, it is expected be high enough to recombine just the genomic *FRT*-like sequence of interest. This assessment is based on the functional properties of the *FRT*-like sequences that are composed of not just two recombinase binding sequences but also of the spacer between these sequences (Figs. 1A, 2B), which plays a critical role in controlling the functional specificity of the recombination reaction (Supplementary Note 1).

In the present study we evolved three Flp variants which served as modules for the Flp-TAL recombinases: FV61, FV63, and FV71 (Fig. 3). These variants have different degree of specificity for their respective target *FRT*-like sequences (Fig. 3D,E): FV63 primarily recombines *FL-63* but also can weakly recombine *FL-61* while FV61 and FV71 are able to recombine all three *FRT*-like sequences: *FL-61*, *FL-63*, and *FL-71*. We evolved FV61, FV63, and FV71 to be only moderately active in bacteria (Fig. 3) since such low activity makes these variants incapable of efficiently targeting genomic *FRT*-like sequences in their native chromosomal environment thus preventing the variants from recombining their targets on their own and thus reducing potential off-target activity of the Flp-TAL recombinases.

For the Flp evolution experiments we developed the two-reporter screening system (Fig. 3A,B), the goal of which is to identify reasonably active recombinase variants with the desired specificity fast, in just one round of protein evolution. Although this system can apparently identify a large number of recombinase variants, the aim of our proof-of-principle experiments was to test its capabilities rather than to use the system to evolve and analyze the properties of a large number of Flp variants.

The profile of mutations in FV61, FV63, and FV71 (Fig. 3C) is different from that of the Flp variants that were evolved previously to recombine the same genomic *FRT*-like sequences^17^. Such variability in the mutational profiles of the target-specific recombinase variants is not unique to Flp^9,36,37^ and is also observed in the target-specific variants of other tyrosine recombinases, such as Cre and λ Int, that were extensively tested for their ability to recombine a variety of the target-like sequences^8,11,21,42–44^. This phenomenon is not surprising considering the lack of the well-defined DNA recognition structures in the tyrosine recombinases^19,20,45^ that apparently utilize both direct and indirect readout of their target sequences. Such target recognition tactic, in turn, translates into a lack of one-to-one correspondence between the amino acid changes and the target specificity. The collective mutational data reveals that amino acid residues, which are modified in the target-specific variants of the tyrosine recombinases, fall primarily into three functional groups: those that contact DNA directly or through water molecules, those that are located in the vicinity of the catalytic residues, and those that are located in the monomer–monomer interfaces. In some recombinase variants, amino acids that do apparently belong to these three groups can also be modified. Among the mutations that are seen in FV61, FV63, and FV71, mutations at positions 55, 58, 59 affect direct DNA binding, while mutations at positions 62, 171, 173, and 285 can affect DNA binding either through direct or indirect interactions. Mutation at position 193 is located next to the catalytic residue Arg-191. Mutations at positons 44, 45, 50, 114, 130 affect monomer–monomer interactions.

Target-specific variants of Flp usually bear a group of mutations that are frequently modified while individual Flp variants have different combinations of these common mutations (Fig. 3C). For example, all three Flp variants that were evolved in this work have a group of common mutations at positions 35, 45, 50, 114, 295, and 363 whereas two of these variants (FV61 and FV63) have a frequent mutation at position 173 and the third variant (FV71) has mutations at the nearby positions: 171 and 176, and only one variant (FV63) has a common mutation at position 324.

Taken together, the mutational profiles of the target-specific variants of the tyrosine recombinases suggest that the entire functional unit of the tyrosine recombinase, which is composed of four DNA-bound monomers^19,20,45^ that form alternating active-inactive pairs^18^, responds to the challenge of recombining target-like DNA sequences by acquiring catalytically competent configuration. Although the catalytically competent configuration that is specific for a particular target-like sequence can be apparently attained by modifying different sets of amino acid residues, it is anticipated that an optimal set can be generated.

In this regard it is worth noting that about 41% of the primary structure of the site-specific tyrosine recombinases SM1 and KW1, which, as Flp, belong to the yeast subfamily of the recombinases, are dissimilar even though the target sequences for these recombinases differ in just one base pair^46^. More than a half of these dissimilar amino acid residues (~ 21.6%) belong to either different or only remotely related chemical types^46^. Although it is expected that not all of the residues that differ between SM1 and KW1 are essential for target recognition, it is still remarkable that the ratio of the dissimilar residues in these recombinases is so high. In all, the sequence analysis of the SM1 and KW1 supports the observation that different sets of mutations, although not necessarily optimal, can help target-specific variants of the tyrosine recombinases to recognize their cognate sequences.

Taking into account the above considerations, we anticipate that Flp variants with the target selection functionality similar to that of FV61, FV63, and FV71 but with different primary sequences can be evolved.

The data in Supplementary Table 1 that shows the average number of colonies of different color obtained in the Flp-TAL integration experiments cannot be easily explained by a single mechanism. Rather, several plausible mechanisms can be proposed. These include: (1) random integration of the targeting vector, (2) site-specific recombination between the targeting vector molecules before they randomly integrate into genome, (3) maintenance of the targeting vector molecules that did not integrate into genome in cells, and (4) site-specific integration of the targeting vector into the desired genome sequence.

Site-specific vector integration, that is the mechanism (4), is inefficient (0.04–0.15%, Fig. 4) and possibly is the least efficient of all four proposed mechanisms. The results on Cre recombinase that were published by Brian Sauer in 1997 suggest that the rate of random vector integration (that is, the mechanism (1)) is ~ tenfold higher than that of site-specific vector integration by tyrosine recombinases^47^. We did not assess the rates of the mechanisms (2) and (3) but we estimate that the rate of the mechanism (2), that is site-specific recombination between the targeting vector molecules, is significant since Flp-TAL recombinases are capable of efficiently recombining their respective vector-borne recognition sequences. The mechanism (2) can lead to various targeting vector multimers with unpredictable expression patterns of the reporter genes. The reasons for such unpredictability reflect complex relationship between active transcription units that is discussed below.

The integration reporter vector that was used in our study contains three transcription units (Fig. 4A). For these transcription units to be expressed approximately at the same level, the nature of their promoters, the location of the units and their relative orientation have to be carefully optimized. This is not a trivial task and we spent a significant amount of time fine-tuning all the necessary parameters. If the transcription units change their relative location/orientation due to recombination between the reporter vector molecules, such an event can alter the expression levels of all three units leading to unique expression patterns. It is reasonable to expect that different Flp-TAL recombinases can generate uniquely rearranged reporter vectors which in turn can lead to colonies with distinctive color profiles. Consequently, we believe that a significant portion of the colonies that are formed in our assays resulted from intermolecular site-specific recombination between vector molecules which were then randomly integrated into genome.

Our targeting reporter vector served its purpose since we were able to obtain site-specific integration positive colonies; nevertheless, the complexity of the associated recombination processes requires further investigation in order to maximize the yield of the desired integration events.

The TAL modules of the Flp-TAL recombinases that were used in our model targeting experiments were programmed to recognize 15-bp long DNA sequences except for the TAL module in the FV71-TAL(L) variant that was programmed to recognize 24-bp long sequence (Fig. 2C). The latter variant was generated since we hypothesized that the longer TAL binding sequence should facilitate more efficient targeting of the *FRT*-like sequences with lower similarity to *FRT*, for example, *FL-71* (Fig. 2B). However, the FV71-TAL(L) variants with the TAL modules that recognize 18-bp and 15-bp long sequences appear to be as efficient as the variant with the TAL module that recognizes 24-bp long sequence (Fig. 4D). Apparently, the Flp-TAL system can function efficiently, at least for the high-scoring *FRT*-like sequences^17^, when all TAL modules in Flp-TAL recognize 15-bp long sequences. These results also make it apparent that the Flp-TAL system should be analyzed to determine what level of dissimilarity between the low-scoring genomic *FRT*-like sequence and *FRT* requires Flp-TAL to utilize the TAL modules that bind to the DNA sequences that are longer than 15 bp to be functional. It is also apparent that the system should be tested in the ‘opposite’ direction: to determine whether the TAL modules that bind to the DNA sequences that are shorter than 15 bp can be still efficient.

In our model genomic target sequences, the TAL recognition sequences are separated from the *FRT*-like sequences by 4–5 bp spacers (Fig. 2C). Further analysis of the Flp-TAL system will determine how the system responds to the shorter or longer spacer sequences.

Potential off-target activity of the Flp-TAL recombinases was analyzed by two approaches. In the first approach, we examined which of the *FRT*-like sequences located on the pTarget reporter is used by a Flp-TAL recombinase with a given specificity to integrate the reporter into the genome. The results of these experiments indicated that the Flp-TAL variants recombine only the *FRT*-like sequences that are specified by their TAL modules (Fig. 5A). In the second approach, we tested whether Flp-TAL delivers pTarget into just the desired genomic *FRT*-like sequence and not into one of the 29 highest-scoring *FRT*-likes sequences that have the same spacer as the intended *FRT*-like sequence (Supplementary Table 4). These experiments were performed with the Flp-TAL variant FV71-TAL61, the FV71 module of which has broad target specificity while its TAL module is specific for the genomic *FRT*-like sequence *FL-61*. Since we did not see any apparent integration of pTarget into these potential ‘off-target’ *FRT*-likes sequences, we conclude that the TAL module of FV71-TAL61 reliably determines which *FRT*-like sequence is targeted. Nevertheless, further research is needed to analyze more potential off-target sequences for FV71-TAL61 and potential off-target activity of other Flp-TAL recombinases.

In contrast to the recCas9 system^7^ that utilizes dCas9 as the target-specific DNA binding module to direct the serine recombinase variant to the desired genome targets, the Flp-TAL system relies on the TAL DNA-binding domain. Although it takes more effort to reprogram the TAL module than the dCas9 module, which can be considered as a disadvantage under some circumstances, the TAL DNA-binding domain provides the Flp-TAL system with an important functional benefit. Since the TAL domain has relaxed requirements for its target selection, the respective cognate sequence can be found predictably close to the recombinase binding sequence to reliably stabilize the recombinase module on its target (Fig. 2B). Consequently, as demonstrated in this work, the activity of the Flp-TAL system is consistently high. In this regard we note that in contrast to the Flp-TAL system, no integration activity was reported for recCas9 on the genomic targets. Moreover, the reduced availability of the potential genomic sequences for the recCas9 system limits its applicability compared to the Flp-TAL system:  ~ 450 potential target sequences in the human genome for recCas9^7^ versus ~ 600,000 *FRT*-like sequences for Flp-TAL^17^.

Recent results from the Buchholz lab demonstrate that heterodimers of the target-specific Cre variants can be used to delete genomic DNA between the *loxP*-like sequences located in the their native chromosomal environment^48^. The heterodimer Cre system does not rely on the extra DNA-binding domains and requires new target-specific Cre variants to be evolved for each *loxP*-like sequence of interest. It is interesting to note that the deletion activity of the heterodimer Cre system and the Flp-TAL system is roughly the same: ~ 36% and ~ 22%, respectively (Ref.^51^, Fig. 6, and Supplementary Fig. 4).

As the tyrosine recombinases have similar three-dimensional organization, similar mode of target binding, and are apparently well amenable to modification of their target specificity, we anticipate that other members of the tyrosine recombinase family can be also used to generate TAL-fused recombinases. These ‘additional’ hybrid recombinases can greatly diversify the sequences that can be targeted by the TAL-fused recombinases since each recombinase has its own set of target-like sequences in a genome. Moreover, different TAL-fused recombinases can be paired to perform dual RMCE to efficiently replace genome fragments. Importantly, the availability of several target-specific hybrid recombinases suitable for dual RMCE would translate into shorter genome fragments that can be replaced: our pilot analysis of the distribution of the target-like sequences for different recombinases in a genome shows that the arsenal of 5–6 hybrid tyrosine recombinases is sufficient for reducing the size of the replaceable genomic fragments to about 1 kb.

## Materials and Methods

### Bacterial experiments

The bacterial experiments were performed in *E. coli* strain NEB 10-beta (New England Biolabs, Ipswich, MA): *araD139 ∆(ara-leu)7697 fhuA lacX74 galK (ϕ80 ∆(lacZ)M15) mcrA galU recA1 endA1 nupG rpsL (StrR) ∆(mrr-hsdRMS-mcrBC*).

The inversion experiments (Fig. 3A), which were designed to identify recombination proficient Flp variants in the initial library of Flp variants, were performed as follows. First, the ligation mixture of the inversion reporter (a derivative of pBAD33^49^) and the Flp variant library was transformed into competent bacterial cells (20 µl) which, after heat shock and subsequent addition of LB medium (0.3 ml), were incubated at 37 °C for 2.5 h with the inducer L-arabinose (0.1%). Then, the transformed cells were transferred into 20 ml of LB supplemented with chloramphenicol (35 µg/ml) and incubated overnight with shacking. The plasmid DNA was then isolated and subjected to the diagnostic PCR to amplify the genes of those Flp variants that were able to invert the reporter cassette.

The deletion experiments (Fig. 3B), which were next applied to identify a subset of the inversion proficient target-specific Flp variants that are sufficiently active to delete the *lacZα* reporter cassette in at least some of the reporter vector molecules, were performed essentially as described in Voziyanov et al.^35^. In brief, the Flp variant gene libraries identified in the deletion experiments were cloned into pBAD33 and transformed into bacterial cells that already harbored the respective deletion reporter vectors (derivatives of pBAD24^49^). The transformed cells were treated with the inducer L-arabinose as described above and then plated onto LB/agar plates which contained X-gal to help identify the colonies that contained cells with the deleted *lacZ*α cassette.

### Flp variant gene libraries

Flp variant gene libraries were generated by shuffling the genes of several target-specific Flp variants: FV7^9^, FV-2798-7, FV61-2, FV-63-n36, FV-71-60^17^, and Flp-sup3^37^. The gene mixture also contained the wild-type Flpe gene^50^ and the Flpe gene library with the randomized codons at positions 55, 58, and 59. Site-specific mutagenesis to randomize Flp codons at positions 55, 58 and 59 was performed as describe earlier^50^ using oligonucleotides that contained all ‘N’s at the respective positions.

The DNA shuffling was performed essentially as described in Bolusani et al.^9^. In brief, the Flp variant genes were first amplified using Taq polymerase (New England Biolabs) and then the resultant PCR products were mixed and fragmented with DNase I. These DNA fragments were reassembled into the Flp gene library by amplifying them first using Pfu–Ultra polymerase (Agilent, Santa Clara, CA) without any specific primers and then by amplifying the resultant PCR products using Taq polymerase with primers that anneal just outside the coding region of the Flp gene. The PCR products of the second amplification (that contained the Flp variant libraries) were cloned into the inversion reporter.

### Experiments in human cells

Human embryonic kidney HEK-293 cells (CRL-1573, ATCC, Manassas, VA) were used as model human cells. These cells were propagated in EMEM medium supplemented with 10% FBS and the antibiotic/antimycotic mixture (penicillin/streptomycin/amphotericin B).

Flp variants and Flp-TAL recombinases were cloned into the pOG100 vector^16^, a derivative of pOG44^31^, in which the CMV promoter is used to drive the expression of the recombinases. The pTarget reporter (Fig. 4A) was constructed using the pDNA3 vector (Invitrogen) as the backbone. To minimize instability of the Flp-TAL recombinases and the pTarget vector variants, their cloning was performed in NEB Stable cells (New England Biolabs).

Turbo293 reagent (Speed BioSystems, Gaithersburg, MD) was used to transfect HEK-293 cells. For transfections, 1/5 of almost confluent cells, which were grown in a well of 24-well plate, were seeded into a new well of the same plate type. Transfections were performed 48 h after seeding cells. We found that such late transfection increases the targeting efficiency of Flp-TAL although the overall transection efficiency drops. The transfection efficiency was estimated by assessing the ratio of red cells to the total number of cells transfected with the reporter pTarget which expresses DsRed (Fig. 4A). Under the specified conditions the transfection efficiency was, on average, ~ 10%.

The experiments to integrate pTarget into *FL-61*, *FL-63*, and *FL-71* (Fig. 4a) were performed as follows. HEK-293 cells were co-transfected, in 24-well plates, with pTarget (0.4 µg) and the respective pOG100-FV-TAL vector (0.4 µg for FV61-TAL and 1 µg for FV63-TAL and FV71-TAL). The indicated amounts of the vectors reflect the best combination identified thus far. 48 h post-transfection, 1/10 of cells were transferred into 6-well plate containing EMEM medium that was supplemented with hygromycin (550 mg/l) 24 h later. The hygromycin resistant colonies that formed were assessed and pooled after about 10–14 days. The genomic DNA from these colonies was isolated and analyzed by PCR and sequencing. Alternatively, individual red colonies (in the FV61-TAL and FV63-TAL experiments) or green colonies (in the FV71-TAL experiments) were transferred into 48-well plate, expanded, and then their genomic DNA was isolated and analyzed by PCR and sequencing.

The deletion experiments were performed in 24-well plates by transfecting 1 µg of pOG100-FV71-TAL(15) into cells that were expanded from the individual integration-positive red colonies obtained in the *FL-61* integration experiments. Transfections in the deletion experiments, as in the integration experiments, were performed 48 h after seeding cells into wells of the 24-well plates. 48 h post transfection, 1/4 of cells were transferred into 6-well plates, allowed to become confluent, collected, their genomic DNA isolated and then analyzed by PCR and sequencing.

The PCR analyses were performed using primer sets shown in Supplementary Table 3.

### Other methods

Plasmid DNA from bacterial cells was isolated using E.Z.N.A. Plasmid Mini Kit I (Omega Bio-tek). Genomic DNA from mammalian cells was isolated using E.Z.N.A. Tissue DNA Kit (Omega Bio-tek). Amplification of the DNA fragments for cloning was performed using Pfu-Ultra polymerase (Agilent). PCR analysis of the genomic DNA was performed using Q5 (New England Biolabs) and GoTag (Promega) master mixes. The ClustalW program (https://www.genome.jp/tools-bin/clustalw) was used for sequence alignments. Golden Gate TALEN kit was used to assemble the DNA binding domains of TAL^28^. General genetic engineering experiments were performed as described in Molecular Cloning Manual^51^. 3D structures of the proteins were analyzed using Swiss-PdbViewer^52^.

## Supplementary information

Supplementary information.

## Data Availability

No datasets were generated or analysed during the current study.

## References

[1] Miller JC (2011). A TALE nuclease architecture for efficient genome editing. Nat. Biotechnol..

[2] Cong L (2013). Multiplex genome engineering using CRISPR/Cas systems. Science.

[3] Komor AC, Kim YB, Packer MS, Zuris JA, Liu DR (2016). Programmable editing of a target base in genomic DNA without double-stranded DNA cleavage. Nature.

[4] Kim YB (2017). Increasing the genome-targeting scope and precision of base editing with engineered Cas9-cytidine deaminase fusions. Nat. Biotechnol..

[5] Anzalone AV (2019). Search-and-replace genome editing without double-strand breaks or donor DNA. Nature.

[6] Grindley ND, Whiteson KL, Rice PA (2006). Mechanisms of site-specific recombination. Annu. Rev. Biochem..

[7] Chaikind B, Bessen JL, Thompson DB, Hu JH, Liu DR (2016). A programmable Cas9-serine recombinase fusion protein that operates on DNA sequences in mammalian cells. Nucl. Acids Res..

[8] Sarkar I, Hauber I, Hauber J, Buchholz F (2007). HIV-1 proviral DNA excision using an evolved recombinase. Science.

[9] Bolusani S (2006). Evolution of variants of yeast site-specific recombinase Flp that utilize native genomic sequences as recombination target sites. Nucl. Acids Res..

[10] Akopian A, He J, Boocock MR, Stark WM (2003). Chimeric recombinases with designed DNA sequence recognition. Proc. Natl. Acad. Sci. U S A.

[11] Buchholz F, Stewart AF (2001). Alteration of Cre recombinase site specificity by substrate-linked protein evolution. Nat. Biotechnol..

[12] Gordley RM, Smith JD, Graslund T, Barbas CF (2007). Evolution of programmable zinc finger-recombinases with activity in human cells. J. Mol. Biol..

[13] Mercer AC, Gaj T, Fuller RP, Barbas CF (2012). Chimeric TALE recombinases with programmable DNA sequence specificity. Nucl. Acids Res..

[14] Skarnes WC (2011). A conditional knockout resource for the genome-wide study of mouse gene function. Nature.

[15] Osterwalder M (2010). Dual RMCE for efficient re-engineering of mouse mutant alleles. Nat. Methods.

[16] Anderson RP, Voziyanova E, Voziyanov Y (2012). Flp and Cre expressed from Flp-2A-Cre and Flp-IRES-Cre transcription units mediate the highest level of dual recombinase-mediated cassette exchange. Nucl. Acids Res..

[17] Shultz JL, Voziyanova E, Konieczka JH, Voziyanov Y (2011). A genome-wide analysis of FRT-like sequences in the human genome. PLoS ONE.

[18] Jayaram M (2015). An overview of tyrosine site-specific recombination: From an Flp perspective. Microbiol. Spectr..

[19] Guo F, Gopaul DN, van Duyne GD (1997). Structure of Cre recombinase complexed with DNA in a site-specific recombination synapse. Nature.

[20] Chen Y, Narendra U, Iype LE, Cox MM, Rice PA (2000). Crystal structure of a Flp recombinase-Holliday junction complex: Assembly of an active oligomer by helix swapping. Mol. Cell.

[21] Karpinski J (2016). Directed evolution of a recombinase that excises the provirus of most HIV-1 primary isolates with high specificity. Nat. Biotechnol..

[22] Shah R, Li F, Voziyanova E, Voziyanov Y (2015). Target-specific variants of Flp recombinase mediate genome engineering reactions in mammalian cells. FEBS J..

[23] Kim YG, Cha J, Chandrasegaran S (1996). Hybrid restriction enzymes: Zinc finger fusions to Fok I cleavage domain. Proc. Natl. Acad. Sci. USA.

[24] Christian M (2010). Targeting DNA double-strand breaks with TAL effector nucleases. Genetics.

[25] Porteus MH, Carroll D (2005). Gene targeting using zinc finger nucleases. Nat. Biotechnol..

[26] Urnov FD (2005). Highly efficient endogenous human gene correction using designed zinc-finger nucleases. Nature.

[27] Miller JC (2007). An improved zinc-finger nuclease architecture for highly specific genome editing. Nat. Biotechnol..

[28] Cermak T (2011). Efficient design and assembly of custom TALEN and other TAL effector-based constructs for DNA targeting. Nucl. Acids Res..

[29] Gaj T, Gersbach CA, Barbas CF (2013). ZFN, TALEN, and CRISPR/Cas-based methods for genome engineering. Trends Biotechnol..

[30] Rinaldi FC, Doyle LA, Stoddard BL, Bogdanove AJ (2017). The effect of increasing numbers of repeats on TAL effector DNA binding specificity. Nucl. Acids Res..

[31] O'Gorman S, Fox DT, Wahl GM (1991). Recombinase-mediated gene activation and site-specific integration in mammalian cells. Science.

[32] Raymond CS, Soriano P (2007). High-efficiency FLP and PhiC31 site-specific recombination in mammalian cells. PLoS ONE.

[33] Patsch C (2010). Engineering cell-permeant FLP recombinase for tightly controlled inducible and reversible overexpression in embryonic stem cells. Stem Cells.

[34] Mak AN, Bradley P, Cernadas RA, Bogdanove AJ, Stoddard BL (2012). The crystal structure of TAL effector PthXo1 bound to its DNA target. Science.

[35] Voziyanov Y, Stewart AF, Jayaram M (2002). A dual reporter screening system identifies the amino acid at position 82 in Flp site-specific recombinase as a determinant for target specificity. Nucl. Acids Res..

[36] Voziyanov Y, Konieczka JH, Stewart AF, Jayaram M (2003). Stepwise manipulation of DNA specificity in Flp recombinase: Progressively adapting Flp to individual and combinatorial mutations in its target site. J. Mol. Biol..

[37] Konieczka JH, Paek A, Jayaram M, Voziyanov Y (2004). Recombination of hybrid target sites by binary combinations of Flp variants: Mutations that foster interprotomer collaboration and enlarge substrate tolerance. J. Mol. Biol..

[38] Lamb BM, Mercer AC, Barbas CF (2013). Directed evolution of the TALE N-terminal domain for recognition of all 5' bases. Nucl. Acids Res..

[39] Binz RL (2019). Identification of novel breakpoints for locus- and region-specific translocations in 293 cells by molecular cytogenetics before and after irradiation. Sci. Rep..

[40] Phan QV, Contzen J, Seemann P, Gossen M (2017). Site-specific chromosomal gene insertion: Flp recombinase versus Cas9 nuclease. Sci. Rep..

[41] Sykes PJ (1992). Quantitation of targets for PCR by use of limiting dilution. Biotechniques.

[42] Rufer AW, Sauer B (2002). Non-contact positions impose site selectivity on Cre recombinase. Nucl. Acids Res..

[43] Tay Y, Ho C, Droge P, Ghadessy FJ (2010). Selection of bacteriophage lambda integrases with altered recombination specificity by in vitro compartmentalization. Nucl. Acids Res..

[44] Siau JW (2015). Directed evolution of lambda integrase activity and specificity by genetic derepression. Prot. Eng. Des. Sel..

[45] Biswas T (2005). A structural basis for allosteric control of DNA recombination by lambda integrase. Nature.

[46] Voziyanova E, Anderson RP, Shah R, Li F, Voziyanov Y (2016). Efficient genome manipulation by variants of site-specific recombinases R and TD. J. Mol. Biol..

[47] Bethke B, Sauer B (1997). Segmental genomic replacement by Cre-mediated recombination: Genotoxic stress activation of the p53 promoter in single-copy transformants. Nucl. Acids Res..

[48] Lansing F (2020). A heterodimer of evolved designer-recombinases precisely excises a human genomic DNA locus. Nucl. Acids Res..

[49] Guzman LM, Belin D, Carson MJ, Beckwith J (1995). Tight regulation, modulation, and high-level expression by vectors containing the arabinose PBAD promoter. J. Bacteriol..

[50] Buchholz F, Angrand PO, Stewart AF (1998). Improved properties of FLP recombinase evolved by cycling mutagenesis. Nat. Biotechnol..

[51] Sambrook J, Russell DW (2001). Molecular Cloning: A Laboratory Manual.

[52] Guex N, Peitsch MC (1997). SWISS-MODEL and the Swiss-PdbViewer: An environment for comparative protein modeling. Electrophoresis.

