# Supplementary material for: Genome targeting by hybrid Flp-TAL recombinases
Source: Sci Rep. 2020 Oct 15;10:17479. doi: 10.1038/s41598-020-74474-2 (PMC7562724; doi:10.1038/s41598-020-74474-2)

Supplementary Information

Genome targeting by hybrid Flp-TAL recombinases

Eugenia Voziyanova[1], Feng Li[1], Riddhi Shah[2], Yuri Voziyanov[1]*

[1] School of Biosciences, Louisiana Tech University, 1 Adams Blvd., Ruston, LA 71272, USA

[2] Present address:
Department of Medicine, Columbia University Medical Center, New York, NY 10032, USA

* To whom correspondence should be addressed:

Yuri Voziyanov
Louisiana Tech University
School of Biological Sciences
1 Adams Blvd.
Ruston, LA 71272
USA
Tel.: +1-318-257-2694
Fax: +1-318-257-4574
E-mail: voziyan@latech.edu

Supplementary Note 1. Target specificity of Flp-TAL recombinases, the Flp module of which can recombine multiple *FRT*-like sequences.

Target specificity of the hybrid Flp-TAL recombinases is the product of target specificity of the TAL and the Flp modules. For the sake of this analysis, we consider target specificity of the TAL modules a fixed value and estimate the overall specificity of Flp-TAL that contain the Flp module with broad target specificity by assessing the probability to find an *FRT*-like sequence between two binding sequences for the TAL modules (see Figs. 1 and 2).

Target specificity of the Flp module that is capable of recombining several *FRT*-like sequences can be estimated based on the sequence characteristics of the *FRT*-like sequences that differ them from random DNA sequences. In mammalian genomes, these sequence characteristics translate into one *FRT*-like sequence per about 5,000 base pairs (Shultz et al., 2011). As such, the probability to find an *FRT*-like sequence between the two sequences for the TAL modules is ~1/5,000. Consequently, the target specificity of Flp-TAL should be about three orders of magnitude higher than that of the TAL modules.

Additionally, we have to consider the important property of the Flp/*FRT* system that depends on the spacer sequence of the recombination target to function: only the targets with the same spacers will efficiently recombine with each other while the targets with different spacers will not. Since *FRT* has an 8-bp spacer, the probability to find a spacer sequence of this length is $1/4^8$ (or 1/65,536). For the high-scoring *FRT*-like sequences this probability is higher since the first and the last base pairs of the spacer in these sequences are invariant: T/A and A/T, respectively, and the G/C content of the spacer is set to be equal or lower than 50% (Shultz et al., 2011). Collectively, these spacer features increase the probability to find two *FRT*-like sequences with the same spacer to $1/4^6/2$ (or ~1/2,000).

Taken together, the theoretical probability to find an *FRT*-like sequence with a unique spacer between two TAL binding sequences is ~1/10^7 (~1/(5x10^3) x ~1/(2x10^3)). Such low probability should ensure that the TAL-guided Flp variant with broad target specificity will recombine just the *FRT*-like sequence of interest. This, of course, can only be realized if the Flp module is not sufficiently active to recombine the *FRT*-like sequences on its own, without being stabilized on the target by the TAL module.

... ATCCTACATAAATAGACGC**ATA** **GGTACC**
GGATCAGGCGGAAGCGGAGGCTCAGGCGGAAGCGGCACATCA
GTGGATCTACGCACGCTCGGCTACAGTC ...

Supplementary Figure 1. Sequence of the fusion between the Flp and the TAL genes.

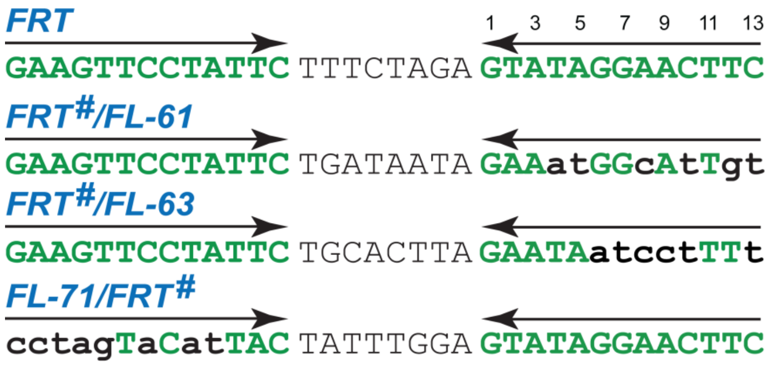

Supplementary Figure 2. Hybrid *FRT*#/*FL-61*, *FRT*#/*FL-63*, and *FL-71/FRT*# sequences. *FRT* is shown above the sequences for comparison.

Colony color	Recombinases			
	**FV61-TAL**	**FV63-TAL**	**FV71-TAL**	**Control**
**Red**	80±33	125±36	94±29	52±22
**Green**	885±201	493±114	295±54	1240±146
**Red/Green**	381±117	750±129	334±39	1100±186
**Colorless**	386±91	325±48	154±48	149±43

Supplementary Table 1. Average number of colony types in the integration experiments performed with the FV61-TAL, FV63-TAL, and FV71-TAL recombinases. Control experiments were performed with the recombinase R1-6 (Voziyanova et al., 2015), which cannot recognize the *FRT*-like sequences in pTarget. The integration experiments were performed in three biological replicates with the same batch of the vectors.

	Recombinases		
	**FV61-TAL**	**FV63-TAL**	**FV71-TAL**
**Integration-positive**	9 (81)	7 (73)	5 (80)
**colonies**	8 (67)	9 (76)	3 (77)

Supplementary Table 2. Fraction of the integration-positive colonies obtained in the FV61-TAL, FV63-TAL, and FV71-TAL experiments. The number of the integration-positive colonies are shown and the total number of the hygromycin-resistant colonies of the respective color analyzed (red in the FV61-TAL and FV63-TAL experiments and green in the FV71-TAL experiments) are given in parenthesis.

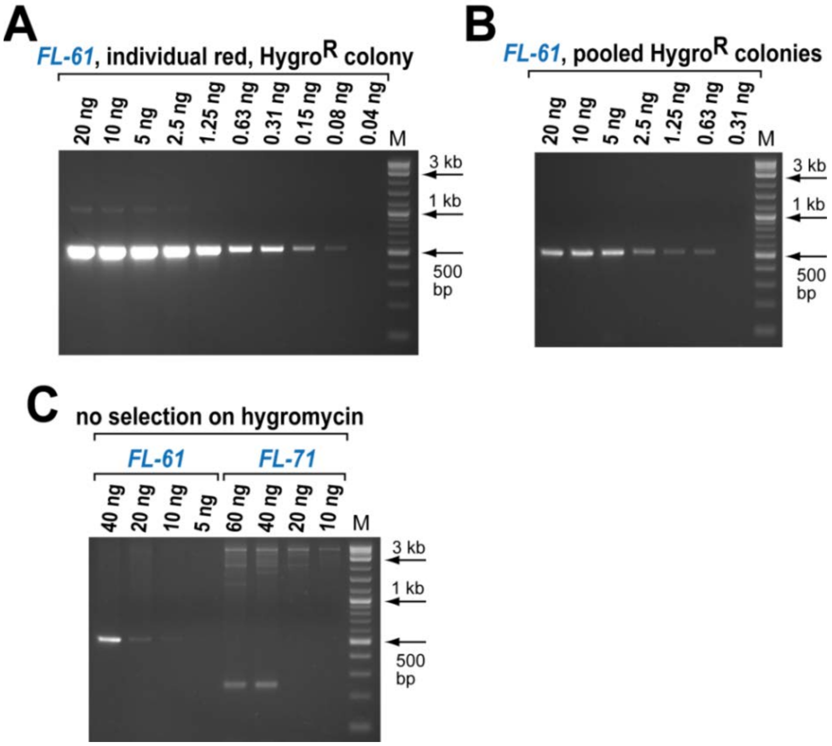

Supplementary Figure 3. PCR-based assessment of the Flp-TAL integration efficiency. PCR analyses of (**A**) genomic DNA isolated from individual hygromycin-resistance, integration-positive colony, (**B**) from the pooled hygromycin-resistance colonies that were generated in the *FL-61* targeting experiments (see Fig. 4), and (**C**) from pooled cells that were co-transfected with pTarget and FV61-TAL and FV71-TAL, respectively, and then expanded in the growth medium without hygromycin. The PCR analyses were performed at the right junction between the integrated pTarget and genome DNA (Fig. 4). M, 2-log DNA ladder (NEB).

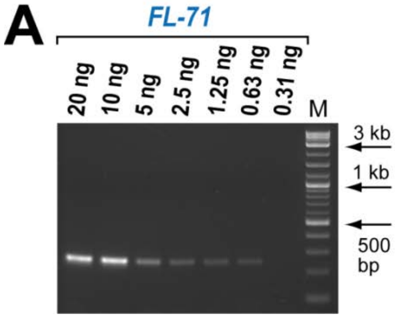

B

Exp 1	Exp 2	Exp 3
0.63 ng	0.63 ng	0.31 ng

Supplementary Figure 4. PCR-based assessment of the deletion activity of FV71-TAL. (**A**) PCR analysis of genomic DNA isolated from the *FL-61* integration-positive cells that were transfected with FV71-TAL (see Fig. 4a). (**B**) The experiments were performed in three biological replicates; the results of these experiments are indicated. The PCR analyses were

performed at the right junction between the integrated pTarget and genome DNA (see Fig. 6b). M, 2-log DNA ladder (NEB).

Supplementary Table 3. Oligonucleotide pairs that were used to PCR amplify control DNA fragments in the targeting experiments in human cells

Region	Oligonucleotide sequence
**Left Junction**	
FL-61	GTATCCAAGACTGTATTTCTGATTTTATCGTACC (anneals at ~90 bp upstream of *FL-61*)<br>CCTCGAACTTCACCTCGGCGCGGGTCTTGTAGTTG (anneals in the middle of the EGFP gene in pTarget)
FL-63	CTGATAGGCACTGACTCTCTGTCCCTTGGGCTG (anneals at ~550 bp upstream of *FL-63*)<br>CCTCGAACTTCACCTCGGCGCGGGTCTTGTAGTTG (anneals in the middle of the EGFP gene in pTarget)
FL-71	GGTTAAGTTCATGTCATAGGAAGGGGATAAGTAACAGGG (anneals at ~280 bp upstream of *FL-71*)<br>GGTGGCGAGGCGCACCGTGGGCTTGTACTCGG (anneals at the beginning of the puro-2A-DsRed gene in pTarget)
**Right Junction**	
FL-61	GGACTTTCCAAAATGTCGTAACAACTCCGC (anneals at the 3'-end of the CMV promoter in pTarget)<br>AAAGATGGATGATGTGCCTGAGATTCTGATCACAAGG (anneals at ~270 bp downstream from *FL-61*)
FL-63	CATCGGCATAGTATATCGGCATAGTATAATACGAC (anneals at the 5'-end of the recombination target cassette I in pTarget)<br>CTTAGACAAAACTGATCCCCAGGTTATTCCCATCAG (anneals at ~490 bp downstream from *FL-63*)
FL-71	GCAAAACCTTTCGCGGTATGGCATGATAGCGCCC (anneals at the 5'-end of the recombination target cassette II in pTarget)<br>CAATATGTGTACACATATTAAAACATTACACTTTAACCC (anneals at ~115 bp downstream from *FL-71*)

Supplementary Table 4. *FRT*-like sequences in the human genome that were tested for potential off-target integration events. The *FRT*-like sequences shown have the highest level of homology to *FRT* (Shultz et al., 2011) and their spacer sequences, which are highlighted in bold, are identical to that of *FL-61*, see Fig. 2b. The relative orientation of these *FRT*-like sequences (which is specified by the direction of their spacer sequences) is different in the human chromosomes but these sequences are arranged in such a way that the direction of their spacers matches that of *FL-61* (Fig. 2b).

***FRT*-like sequence**
AACAATCCTATTC**TGATAATA**AAATAAAGCTGAT, **Chr-01**
TATTATCATATTT**TGATAATA**AAATATGATTTGA, **Chr-01**
AGTTTTCCAATAT**TGATAATA**GTATAAACAAGCA, **Chr-01**
GATTGGGCAATTT**TGATAATA**AAATAAGAATTCC, **Chr-02**
ATGTTTCTGATAC**TGATAATA**GTATAAGTGCCAA, **Chr-02**
AGTTCGCAAATTC**TGATAATA**GAATTGCTCCTCT, **Chr-03**
AGAATAATTATTC**TGATAATA**ATATTAGTAATAA, **Chr-03**
AATTTTCTTATTT**TGATAATA**GTATAATGGTTAT, **Chr-03**
TAAGTGAATATTT**TGATAATA**AAATTGGAGATCA, **Chr-04**
GCTTGCCTGATTC**TGATAATA**ATATTCAAGCAAA, **Chr-04**
AGTTTTCTTATAT**TGATAATA**AAATTGTTAATGT, **Chr-04**
GTTTCACATATAT**TGATAATA**GTATCACCATATC, **Chr-06**
TGACTTTTGATAC**TGATAATA**GAATAAGCACATT, **Chr-06**
ACCAGTATGATTC**TGATAATA**GAATAGGTTGAGT, **Chr-08**
ACCAGTATCATTT**TGATAATA**AAATCTGACAGAG, **Chr-11**
TGCAAACAGATAC**TGATAATA**GAATATGAAATAA, **Chr-11**
CACCAATTGATAC**TGATAATA**AAATATGTTCATT, **Chr-11**
TTTAGTATAATTT**TGATAATA**AAATAAGAGTAAT, **Chr-12**
TTTTCCAATATAT**TGATAATA**AAATAGGTTTGCT, **Chr-12**

GATTTCCTAATTT**TGATAATA**GTATAGTGCTTAT, **Chr-13**

CAGTAAATGATAT**TGATAATA**GTATAAGTACTGT, **Chr-14**

GTCTGTCATATTC**TGATAATA**ATATGATTTAATA, **Chr-14**

AAGACATCAATAC**TGATAATA**GTATATGATGTTA, **Chr-14**

AATATACTCATTT**TGATAATA**AAATATAGTATGC, **Chr-18**

TTGATATTAATAT**TGATAATA**ATATCAGTATGTC, **Chr-21**

AGATAACCAATAT**TGATAATA**ATATAATAATCAT, **Chr-X**

ATCTCTCTCATAT**TGATAATA**ATATATCAATTGG, **Chr-X**

CATCAATTTATAC**TGATAATA**AAATTTGAATTTT, **Chr-X**

TATGGGATCATAC**TGATAATA**GTATAAGGGTTCG, **Chr-Y**

pTarget vector sequence (without recombination targets)

GACGGATCGGGAGATCTCCCGATCCCCTATGGTCGACTCTCAGTACAATCTGCTCTGATGCCGCATAGTT
AAGCCAGTATCTGCTCCCTGCTTGTGTGTTGGAGGTCGCTGAGTAGTGCGCGAGCAAAATTTAAGCTACA
ACAAGGCAAGGCTTGACCGACAATTGCATGAAGAATCTGCTTAGGGTTAGGCGTTTTGCGCTGCTTCGCG
ATGTACGGGCCAGATATACGCGTTGACATTGATTATTGACTAGTTATTAATAGTAATCAATTACGGGGTC
ATTAGTTCATAGCCCATATATGGAGTTCCGCGTTACATAACTTACGGTAAATGGCCCGCCTGGCTGACCG
CCCAACGACCCCCGCCCATTGACGTCAATAATGACGTATGTTCCCATAGTAACGCCAATAGGGACTTTCC
ATTGACGTCAATGGGTGGACTATTTACGGTAAACTGCCCACTTGGCAGTACATCAAGTGTATCATATGCC
AAGTACGCCCCCTATTGACGTCAATGACGGTAAATGGCCCGCCTGGCATTATGCCCAGTACATGACCTTA
TGGGACTTTCCTACTTGGCAGTACATCTACGTATTAGTCATCGCTATTACCATGGTGATGCGGTTTTGGC
AGTACATCAATGGGCGTGGATAGCGGTTTGACTCACGGGGATTTCCAAGTCTCCACCCCATTGACGTCAA
TGGGAGTTTGTTTTGGCACCAAAATCAACGGGACTTTCCAAAATGTCGTAACAACTCCGCCCCATTGACG
CAAATGGGCGGTAGGCGTGTACGGTGGGAGGTCTATATAAGCAGAGCTCTCTGGCTAACTAGAGAACCCA
CTGCTTACTGGCTTATCGAAATTAATACGACTCACTATAGGGAGACCC**AAGCTT**
Recombination target cassette I
CTTCcctttgaaaaacacgatgataatatggccacaacc**ATG**GTGAGCAAGGGCGAGGAGCTGTTCACCG
GGGTGGTGCCCATCCTGGTCGAGCTGGACGGCGACGTAAACGGCCACAAGTTCAGCGTGTCCGGCGAGGG
CGAGGGCGATGCCACCTACGGCAAGCTGACCCTGAAGTTCATCTGCACCACCGGCAAGCTGCCCGTGCCC
TGGCCCACCCTCGTGACCACCCTGACCTACGGCGTGCAGTGCTTCAGCCGCTACCCCGACCACATGAAGC
AGCACGACTTCTTCAAGTCCGCCATGCCCGAAGGCTACGTCCAGGAGCGCACCATCTTCTTCAAGGACGA
CGGCAACTACAAGACCCGCGCCGAGGTGAAGTTCGAGGGCGACACCCTGGTGAACCGCATCGAGCTGAAG
GGCATCGACTTCAAGGAGGACGGCAACATCCTGGGGCACAAGCTGGAGTACAACTACAACAGCCACAACG

TCTATATCATGGCCGACAAGCAGAAGAACGGCATCAAGGTGAACTTCAAGATCCGCCACAACATCGAGGA
CGGCAGCGTGCAGCTCGCCGACCACTACCAGCAGAACACCCCCATCGGCGACGGCCCCGTGCTGCTGCCC
GACAACCACTACCTGAGCACCCAGTCCGCCCTGAGCAAAGACCCCAACGAGAAGCGCGATCACATGGTCC
TGCTGGAGTTCGTGACCGCCGCCGGGATCACTCTCGGCATGGACGAGCTGTACAAG**TAA**AGCGGCCGCGA
CTCTAGATCATAATCAGCCATACCACCTCGAGCATGCATCTAGAGGGCCCTATTCTATAGTGTCACCTAA
ATGCTAGAGCTCGCTGATCAGCCTCGACTGTGCCTTCTAGTTGCCAGCCATCTGTTGTTTGCCCCTCCCC
CGTGCCTTCCTTGACCCTGGAAGGTGCCACTCCCACTGTCCTTTCCTAATAAAATGAGGAAATTGCATCG
CATTGTCTGAGTAGGTGTCATTCTATTCTGGGGGGTGGGGTGGGGCAGGACAGCAAGGGGGAGGATTGGG
AAGACAATAGCAGGCATGCTGGGGATGCGGTGGGCTCTATGGCTTCTGAGGCGGAAAGAACCAGCTGGGG
CTCTAGGGGGTATCCCCACGCGCCCTGTAGCGGCGCATTAAGCGCGGCGGGTGTGGTGGTTACGCGCAGC
GTGACCGCTACACTTGCCAGCGCCCTAGCGCCCGCTCCTTTCGCTTTCTTCCCTTCCTTTCTCGCCACGT
TCGCCGGCTTTCCCCGTCAAGCTCTAAATCGGGGCATCCCTTTAGGGTTCCGATTTAGTGCTTTACGGCA
CCTCGACCCCAAAAAACTTGATTAGGGTGATGGTTCACGTAGTGGGCCATCGCCCTGATAGACGGTTTTT
CGCCCTTTGACGTTGGAGTCCACGTTCTTTAATAGTGGACTCTTGTTCCAAACTGGAACAACACTCAACC
CTATCTCGGTCTATTCTTTTGATTTATAAGGGATTTTGGGGATTTCGGCCTATTGGTTAAAAAATGAGCT
GATTTAACAAAAATTTAACGCGAATTAATTCTGTGGAATGTGTGTCAGGTACCGTTTTATTCTGTCTTTT
TATTGgcttctgatggaattagaacttggcaaaacaatactgagaatgaagtgtatgtggaacagaggct
gctgatctcgttcttcaggctatgaaactgacacatttggaaaccacagtacttagaaccacaaagtggg
aatcaagagaaaaacaatgatcccacgagagatctatagatctatagatcatgagtgggaggaatgagct
ggcccttaatttggttttgcttgtttaaattatgatatccaactatgaaacattatcataaagcaatagt
aaagagccttcagtaaagagcaggcatttatctaatcccaccccacccccacccccgtagctccaatcct
tccattcaaaatgtaggtactctgttctcacccttcttaacaaagtatgacaggaaaaacttccattta
gtggacatctttattgtttaatagatcatcaatttctgcagacttacagcggatcccc**CTA**TTCCTTTGC
CCTCGGACGAGTGCTGGGGCGTCGGTTTCCACTATCGGCGAGTACTTCTACACAGCCATCGGTCCAGACG
GCCGCGCTTCTGCGGGCGATTTGTGTACGCCCGACAGTCCCGGCTCCGGATCGGACGATTGCGTCGCATC
GACCCTGCGCCCAAGCTGCATCATCGAAATTGCCGTCAACCAAGCTCTGATAGAGTTGGTCAAGACCAAT
GCGGAGCATATACGCCCGGAGCCGCGGCGATCCTGCAAGCTCCGGATGCCTCCGCTCGAAGTAGCGCGTC
TGCTGCTCCATACAAGCCAACCACGGCCTCCAGAAGAAGATGTTGGCGACCTCGTATTGGGAATCCCCGA
ACATCGCCTCGCTCCAGTCAATGACCGCTGTTATGCGGCCATTGTCCGTCAGGACATTGTTGGAGCCGAA
ATCCGCGTGCACGAGGTGCCGGACTTCGGGGCAGTCCTCGGCCCAAAGCATCAGCTCATCGAGAGCCTGC
GCGACGGACGCACTGACGGTGTCGTCCATCACAGTTTGCCAGTGATACACATGGGGATCAGCAATCGCGC
ATATGAAATCACGCCATGTAGTGTATTGACCGATTCCTTGCGGTCCGAATGGGCCGAACCCGCTCGTCTG
GCTAAGATCGGCCGCAGCGATCGCATCCATGGCCTCCGCGACCGGCTGCAGAACAGCGGGCAGTTCGGTT
TCAGGCAGGTCTTGCAACGTGACACCCTGTGCACGGCGGGAGATGCAATAGGTCAGGCTCTCGCTGAATT
CCCCAATGTCAAGCACTTCCGGAATCGGGAGCGCGGCCGATGCAAAGTGCCGATAAACATAACGATCTTT
GTAGAAACCATCGGCGCAGCTATTTACCCGCAGGACATATCCACGCCCTCCTACATCGAAGCTGAAAGCA
CGAGATTCTTCGCCCTCCGAGAGCTGCATCAGGTCGGAGACGCTGTCGAACTTTTCGATCAGAAACTTCT
CGACAGACGTCGCGGTGAGTTCAGGCTTTTTGGC**CAT**ggtggcggcgattgcaggtcgaaaggcccggag
atgaggaagaggagaacagcgcggcagacgtgcgcttttgaagcgtgcagaatgccgggcctccggagga
ccttcgggcgcccgccccgcccctgagcccgcccctgagcccgcccccggacccaccccttcccagcctc
tgagcccagaaagcgaaggagcaaagctgctattggccgctgccccaaaggcctacccgcttccattgct
cagcggtgctgtccatctgacgagactagtgagacgtgctacttccatttgtcacgtcctacgacgcgag
ctgcggggcgggggggaacttcctgactaggggaggagagaaggtggcgcgaaggggccaccaaagaacg
gagccggttggcgcctaccggtggatgtggaatgtgtgcgaggccaaggccacttgtgtagcgccaagtg
ccagcggggctgctaagcgcatgctcagactgccttggaaaagcgcctcccctacccggtaCCTAGGtgc
aaagatggataaagttttaaacagagaggaatctttgcagctaatggaccttctaggtcttgaaaggagt
gcctcgtgaggctccggtgcccgtcagtgggcagagcgcacatcgcccacagtccccgagaagttggggg
gaggggtcggcaattgaaccggtgcctagagaaggtggcgcggggtaaactgggaaagtgatgtcgtgta
ctggctccgcctttttcccgagggtgggggagaaccgtatataagtgcagtagtcgccgtgaacgttctt
tttcgcaacgggtttgccgccagaacacaggtaagtgccgtgtgtggttcccgcgggcctggcctcttta
cgggttatggcccttgcgtgccttgaattacttccacctggctgcagtacgtgattcttgatcccgagct
tcgggttggaagtgggtgggagagttcgaggccttgcgcttaaggagccccttcgcctcgtgcttgagtt

gaggcctggcctggcgctggggccgccgcgtgcgaatctggtggcaccttcgcgcctgtctcgctgctt
tcgataagtctctagccatttaaaatttttgatgacctgctgcgacgctttttttctggcaagatagtct
tgtaaatgcgggccaagatctgcacactggtatttcggtttttggggccgcgggcggcgacggggcccgt
gcgtcccagcgcacatgttcggcgaggcggggcctgcgagcgcggccaccgagaatcggacgggggtagt
ctcaagctggccggcctgctctggtgcctggcctcgcgccgccgtgtatcgccccgccctgggcggcaag
gctggcccggtcggcaccagttgcgtgagcggaaagatggccgcttcccggccctgctgcagggagctca
aaatggaggacgcggcgctcgggagagcgggcgggtgagtcacccacacaaaggaaaagggcctttccgt
cctcagccgtcgcttcatgtgactccacggagtaccgggcgccgtccaggcacctcgattagttctcgag
cttttggagtacgtcgtctttaggttggggggaggggttttatgcgatggagtttccccacactgagtgg
gtggagactgaagttaggccagcttggcacttgatgtaattctccttggaatttgccctttttgagtttg
gatcttggttcattctcaagcctcagacagtggttcaaagtttttttcttccatttcaggtgtcgtgagg
aattagcttggtactaatacgactcactatagggagacccaagctggctagg

Recombination target cassette II

GCTAGCATGaccgagtacaagcccacggtgcgcctcgccacccgcgacgacgtcccccgggccgtacgca
ccctcgccgccgcgttcgccgactaccccgccacgcgccacaccgtcgacccggaccgccacatcgagcg
ggtcaccgagctgcaagaactcttcctcacgcgcgtcgggctcgacatcggcaaggtgtgggtcgcggac
gacggcgccgcggtggcggtctggaccacgccggagagcgtcgaagcgggggcggtgttcgccgagatcg
gcccgcgcatggccgagttgagcggttcccggctggccgcgcagcaacagatggaaggcctcctggcgcc
gcaccggcccaaggagcccgcgtggttcctggccaccgtcggcgtctcgcccgaccaccagggcaagggt
ctgggcagcgccgtcgtgctccccggagtggaggcggccgagcgcgccggggtgcccgccttcctggaga
cctccgcgccccgcaacctccccttctacgagcggctcggcttcaccgtcaccgccgacgtcgaggtgcc
cgaaggaccgcgcacctggtGCATGACCCGCAAGCCCGGTGCCTCTAGAAAAATTGTCGCTCCTGTCAAA
CAAACTCTTAACTTTGATTTACTCAAACTGGCTGGGGATGTAGAAAGCAATCCAGGGCCCGCCTCCTCCG
AGGACGTCATCAAGGAGTTCATGCGCTTCAAGGTGCGCATGGAGGGCTCCGTGAACGGCCACGAGTTCGA
GATCGAGGGCGAGGGCGAGGGCCGCCCCTACGAGGGCACCCAGACCGCCAAGCTGAAGGTGACCAAGGGC
GGCCCCCTGCCCTTCGCCTGGGACATCCTGTCCCCCCAGTTCCAGTACGGCTCCAAGGTGTACGTGAAGC
ACCCCGCCGACATCCCCGACTACAAGAAGCTGTCCTTCCCCGAGGGCTTCAAGTGGGAGCGCGTGATGAA
CTTCGAGGACGGCGGCGTGGTGACCGTGACCCAGGACTCCTCCCTcCAGGACGGCTCCTTCATCTACAAG
GTGAAGTTCATCGGCGTGAACTTCCCCTCCGACGGCCCCGTAATGCAGAAGAAGACTATGGGCTGGGAGG
CCTCCACCGAGCGCCTGTACCCCCGCGACGGCGTGCTGAAGGGCGAGATCCACAAGGCCCTGAAGCTGAA
GGACGGCGGCCACTACCTGGTGGAGTTCAAGTCTATCTATATGGCCAAGAAGCCCGTGCAGCTGCCCGGC
TACTACTACGTGGACTCCAAGCTGGACATCACCTCCCACAACGAGGACTACACCATCGTGGAGCAGTACG
AGCGCGCCGAGGGCCGCCACCACCTGTTCCTG**TAA**GGATCCGCGGGACTCTGGGGTTCGAAATGACCGAC
CAAGCGACGCCCAACCTGCCATCACGAGATTTCGATTCCACCGCCGCCTTCTATGAAAGGTTGGGCTTCG
GAATCGTTTTCCGGGACGCCGGCTGGATGATCCTCCAGCGCGGGGATCTCATGCTGGAGTTCTTCGCCCA
CCCCAACTTGTTTATTGCAGCTTATAATGGTTACAAATAAAGCAATAGCATCACAAATTTCACAAATAAA
GCATTTTTTTCACTGCATTCTAGTTGTGGTTTGTCCAAACTCATCAATGTATCTTATCATGTCTGTATAC
CGTCGACCTCTAGCTAGAGCTTGGCGTAATCATGGTCATAGCTGTTTCCTGTGTGAAATTGTTATCCGCT
CACAATTCCACACAACATACGAGCCGGAAGCATAAAGTGTAAAGCCTGGGGTGCCTAATGAGTGAGCTAA
CTCACATTAATTGCGTTGCGCTCACTGCCCGCTTTCCAGTCGGGAAACCTGTCGTGCCAGCTGCATTAAT
GAATCGGCCAACGCGCGGGGAGAGGCGGTTTGCGTATTGGGCGCTCTTCCGCTTCCTCGCTCACTGACTC
GCTGCGCTCGGTCGTTCGGCTGCGGCGAGCGGTATCAGCTCACTCAAAGGCGGTAATACGGTTATCCACA
GAATCAGGGGATAACGCAGGAAAGAACATGTGAGCAAAAGGCCAGCAAAAGGCCAGGAACCGTAAAAAGG
CCGCGTTGCTGGCGTTTTTCCATAGGCTCCGCCCCCCTGACGAGCATCACAAAAATCGACGCTCAAGTCA
GAGGTGGCGAAACCCGACAGGACTATAAAGATACCAGGCGTTTCCCCCTGGAAGCTCCCTCGTGCGCTCT
CCTGTTCCGACCCTGCCGCTTACCGGATACCTGTCCGCCTTTCTCCCTTCGGGAAGCGTGGCGCTTTCTC
AATGCTCACGCTGTAGGTATCTCAGTTCGGTGTAGGTCGTTCGCTCCAAGCTGGGCTGTGTGCACGAACC
CCCCGTTCAGCCCGACCGCTGCGCCTTATCCGGTAACTATCGTCTTGAGTCCAACCCGGTAAGACACGAC
TTATCGCCACTGGCAGCAGCCACTGGTAACAGGATTAGCAGAGCGAGGTATGTAGGCGGTGCTACAGAGT
TCTTGAAGTGGTGGCCTAACTACGGCTACACTAGAAGGACAGTATTTGGTATCTGCGCTCTGCTGAAGCC
AGTTACCTTCGGAAAAAGAGTTGGTAGCTCTTGATCCGGCAAACAAACCACCGCTGGTAGCGGTGGTTTT
TTTGTTTGCAAGCAGCAGATTACGCGCAGAAAAAAAGGATCTCAAGAAGATCCTTTGATCTTTTCTACGG

GGTCTGACGCTCAGTGGAACGAAAACTCACGTTAAGGGATTTTGGTCATGAGATTATCAAAAAGGATCTT
CACCTAGATCCTTTTAAATTAAAAATGAAGTTTTAAATCAATCTAAAGTATATATGAGTAAACTTGGTCT
GACAGTTACCAATGCTTAATCAGTGAGGCACCTATCTCAGCGATCTGTCTATTTCGTTCATCCATAGTTG
CCTGACTCCCCGTCGTGTAGATAACTACGATACGGGAGGGCTTACCATCTGGCCCCAGTGCTGCAATGAT
ACCGCGAGACCCACGCTCACCGGCTCCAGATTTATCAGCAATAAACCAGCCAGCCGGAAGGGCCGAGCGC
AGAAGTGGTCCTGCAACTTTATCCGCCTCCATCCAGTCTATTAATTGTTGCCGGGAAGCTAGAGTAAGTA
GTTCGCCAGTTAATAGTTTGCGCAACGTTGTTGCCATTGCTACAGGCATCGTGGTGTCACGCTCGTCGTT
TGGTATGGCTTCATTCAGCTCCGGTTCCCAACGATCAAGGCGAGTTACATGATCCCCCATGTTGTGCAAA
AAAGCGGTTAGCTCCTTCGGTCCTCCGATCGTTGTCAGAAGTAAGTTGGCCGCAGTGTTATCACTCATGG
TTATGGCAGCACTGCATAATTCTCTTACTGTCATGCCATCCGTAAGATGCTTTTCTGTGACTGGTGAGTA
CTCAACCAAGTCATTCTGAGAATAGTGTATGCGGCGACCGAGTTGCTCTTGCCCGGCGTCAATACGGGAT
AATACCGCGCCACATAGCAGAACTTTAAAAGTGCTCATCATTGGAAAACGTTCTTCGGGGCGAAAACTCT
CAAGGATCTTACCGCTGTTGAGATCCAGTTCGATGTAACCCACTCGTGCACCCAACTGATCTTCAGCATC
TTTTACTTTCACCAGCGTTTCTGGGTGAGCAAAAACAGGAAGGCAAAATGCCGCAAAAAAGGGAATAAGG
GCGACACGGAAATGTTGAATACTCATACTCTTCCTTTTTCAATATTATTGAAGCATTTATCAGGGTTATT
GTCTCATGAGCGGATACATATTTGAATGTATTTAGAAAAATAAACAAATAGGGGTTCCGCGCACATTTCC
CCGAAAAGTGCCACCTGACGTC

Recombination target cassettes I

FRT#/FL-61

CATCGGCATAGTATATCGGCATAGTATAATACGACAAGGTGAGGAACTAAACC
CCTATTCTTGTGTTCACGACTGACATC**A**CCGT GAAGTTCCTATAC **TGATAATA**
GAAat**GG**c**A**t**T**gt CACTT**T**CTTCCCTACTGCAACAGAAGCCCAGCT

FRT#/FL-63

CATCGGCATAGTATATCGGCATAGTATAATACGACAAGGTGAGGAACTAAACC
ACAACTCAAATTATGCATACC**A**GCTCT GAAGTTCCTATAC **TGCACTTA** GAATAATCCTTTT
GTCTC**T**CCACATGGGTATGGGAGAGGC

Recombination target cassette II

FL-71/FRT#

tggtgcaaaacctttcgcggtatggcatgatagcgcccggaagagagtcaattcagg
GAGATACATTAAGTAACTTAAAAAAAAACTTTACAC**A**GTCTG cctag**T**a**C**at**TAC**
TATTTGGA GTATAGGAACTTC TATT**T**GCATATTCATAATCTCCCTACTTTATTTTCT

Supplementary Figure 5. Partial sequencing traces of the diagnostic PCR bands shown in Figures 4B and 6B. (**A**) and (**B**), FV61-TAL dependent integration of pTarget into *FL-61;* LJ and RJ, respectively. (**C**) and (**D**), FV63-TAL dependent integration of pTarget into *FL-63;* LJ and RJ, respectively. (**E**) and (**F**), FV71-TAL dependent integration of pTarget into *FL-71;* LJ and RJ, respectively. (**G**) FV71-TAL dependent deletion of pTarget and genomic sequences*;* RJ, respectively.

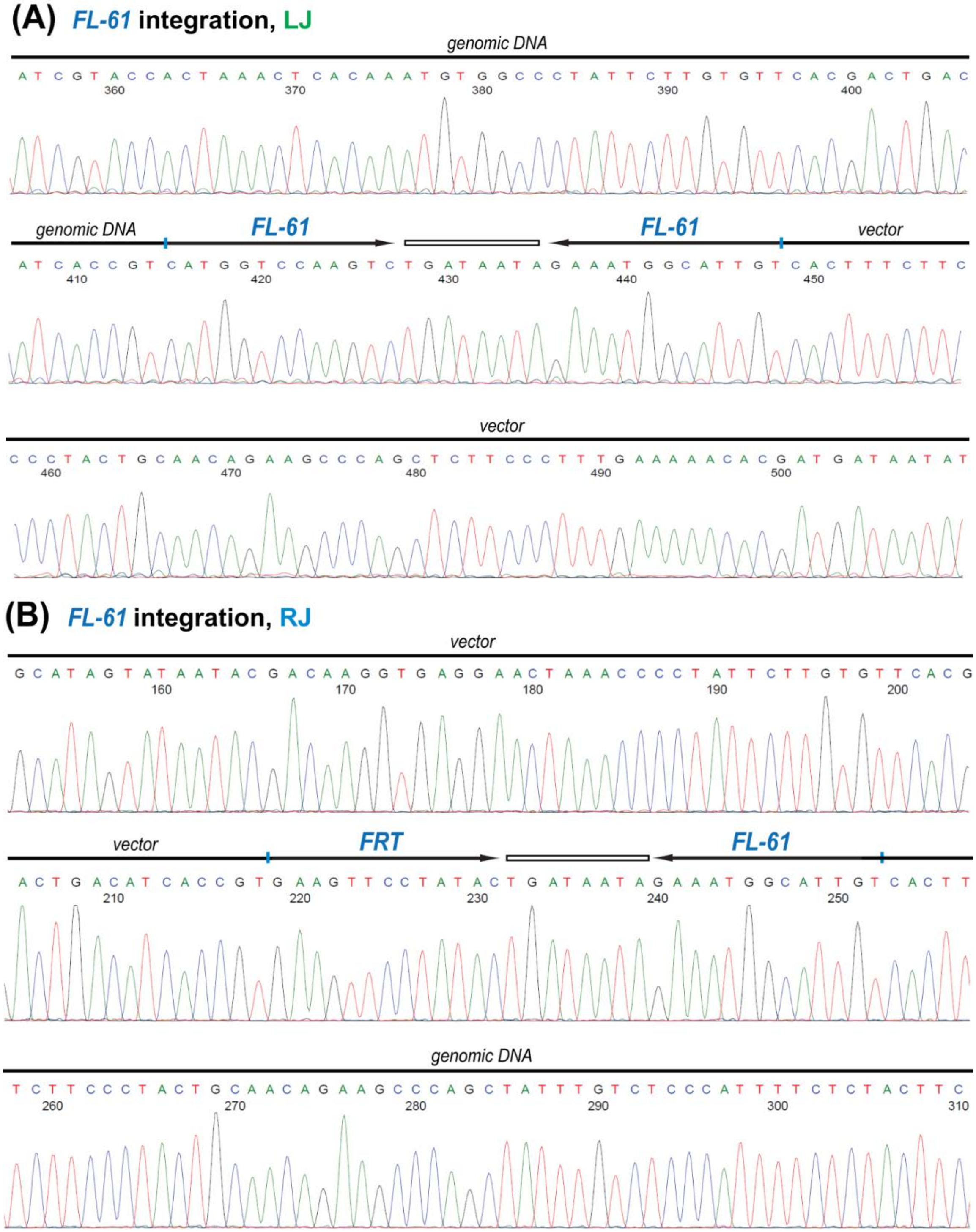

(C) *FL-63* integration, LJ

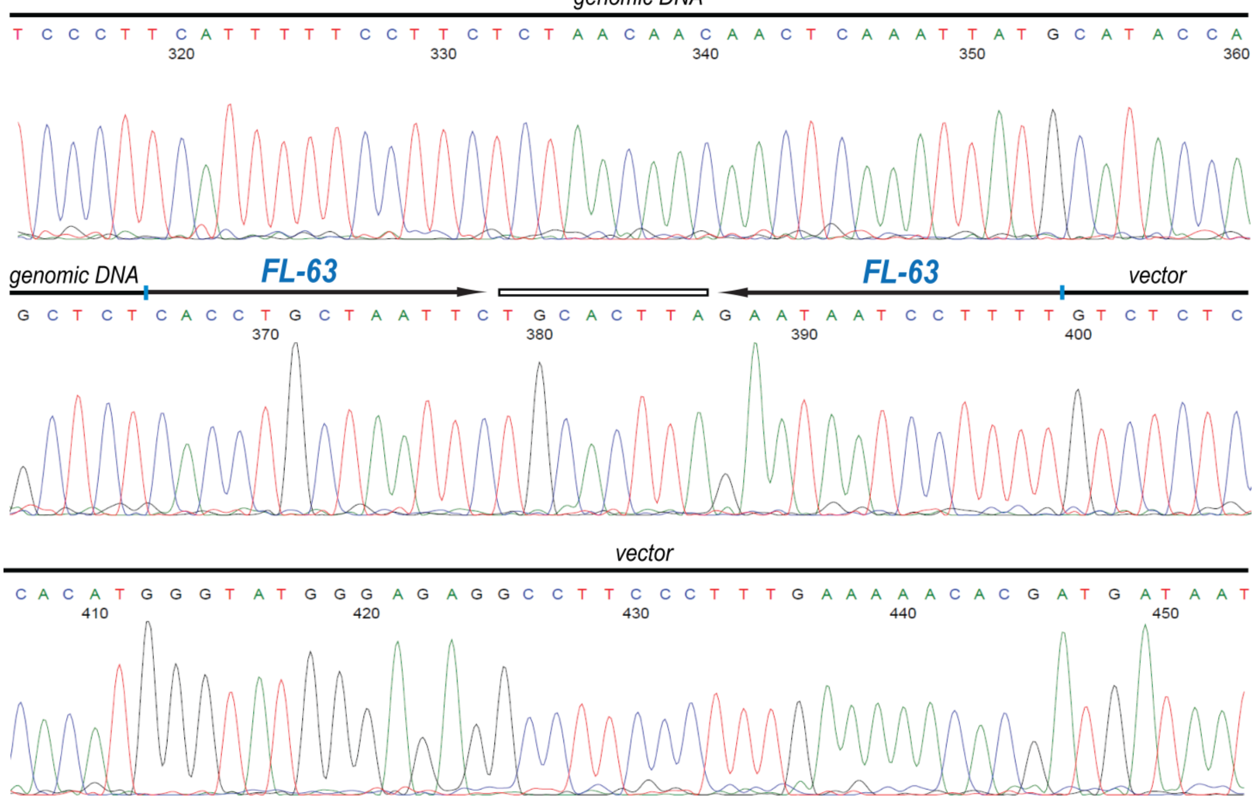

(D) *FL-63* integration, RJ

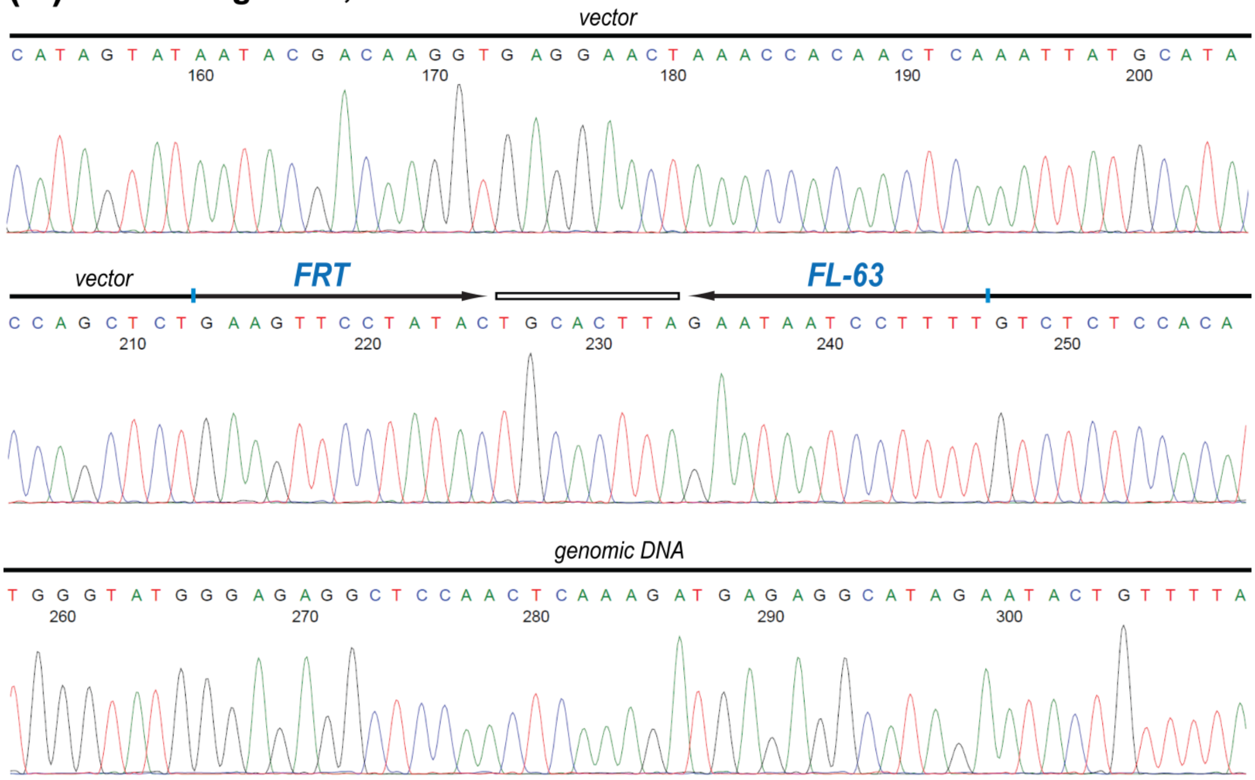

(E) *FL-71* integration, LJ

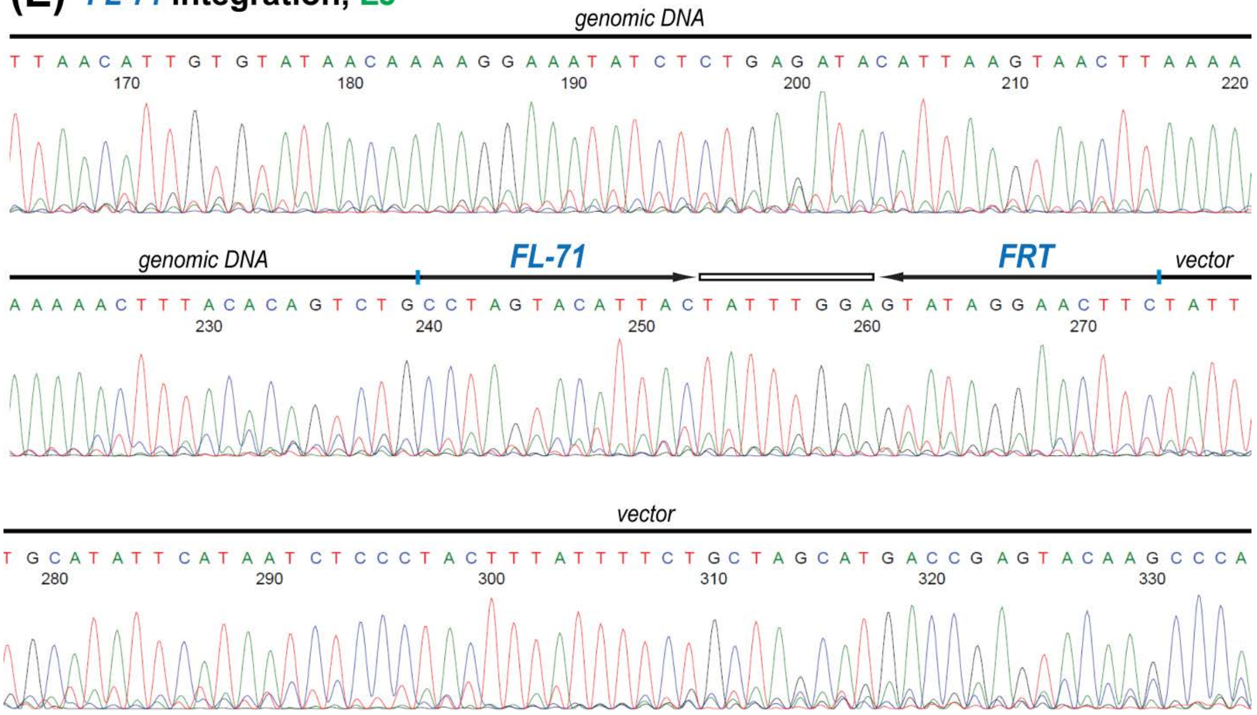

(F) *FL-71* integration, RJ

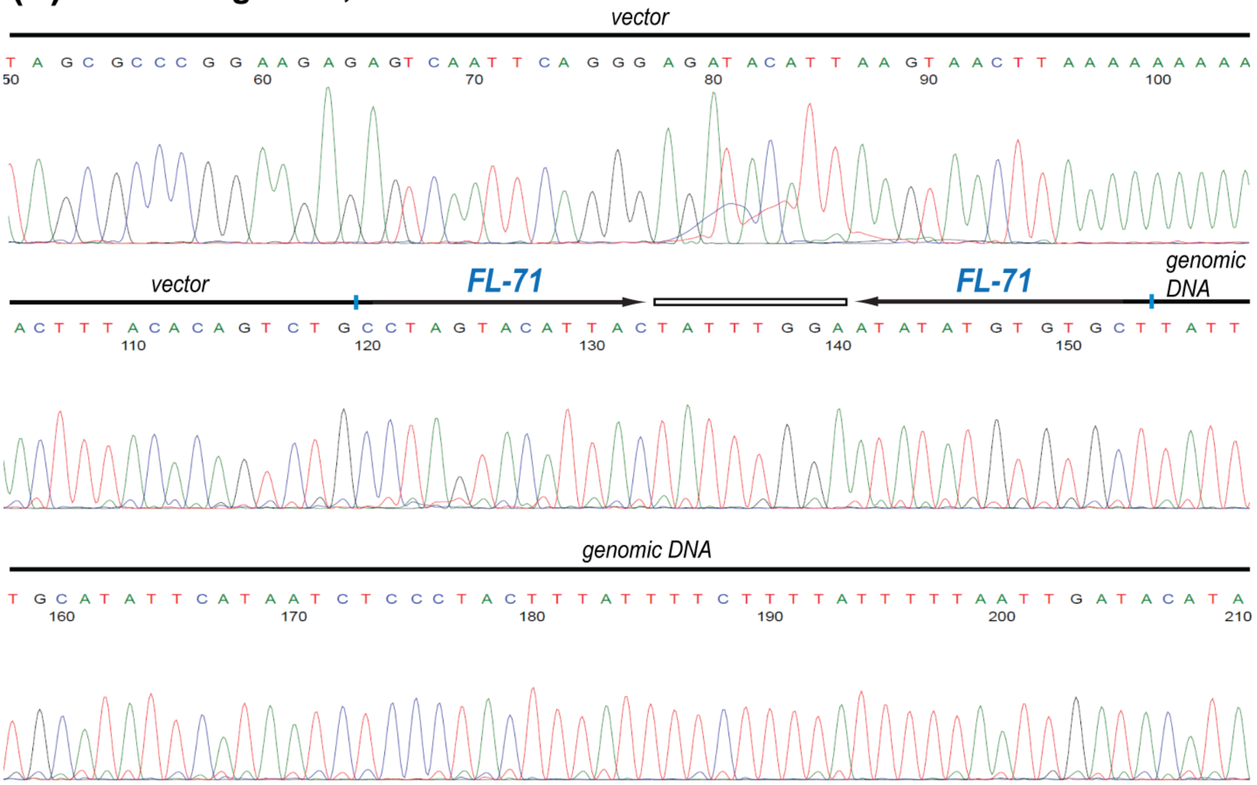

(G) *FL-71* deletion, RJ

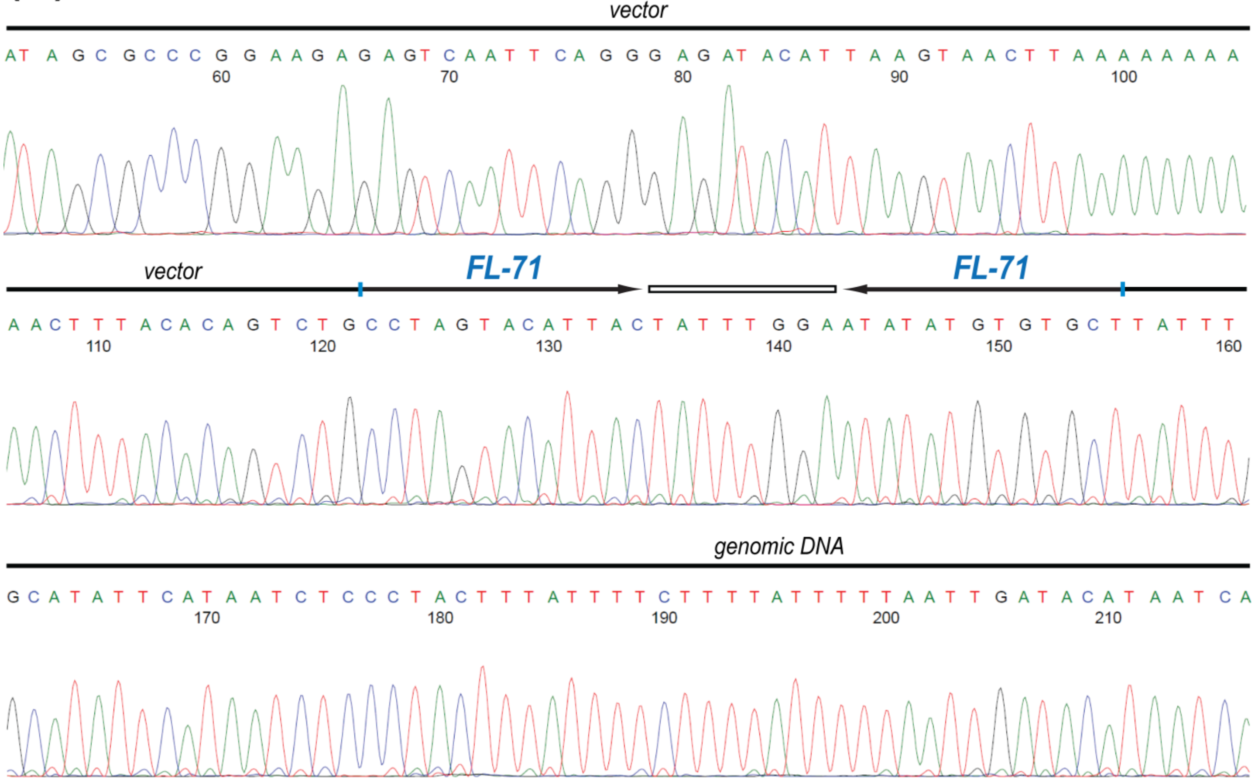

Supplementary Figure 6. Original images of the gels.

Figure 3E.

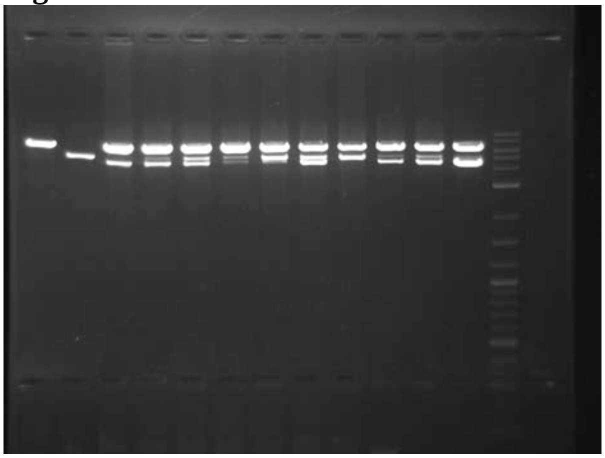

Figure 4B.

LJ

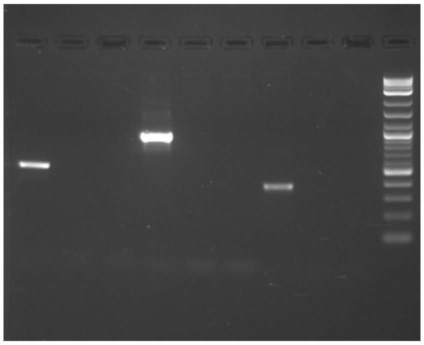

RJ

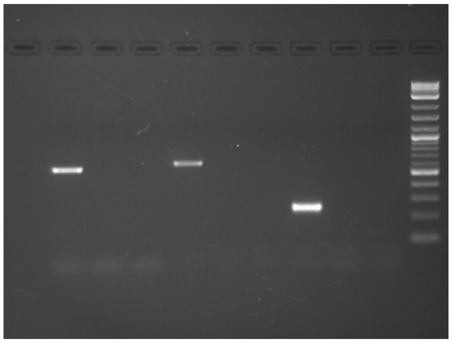

Figure 4D.

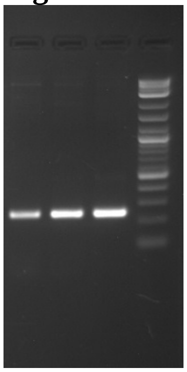

Figure 5A and B.

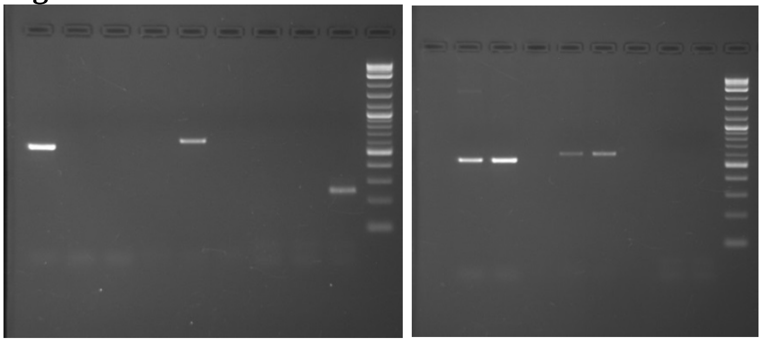

Figure 6B.

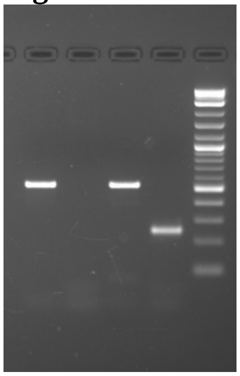

Supplementary Figure 3A, B, and C.

A **B** **C**

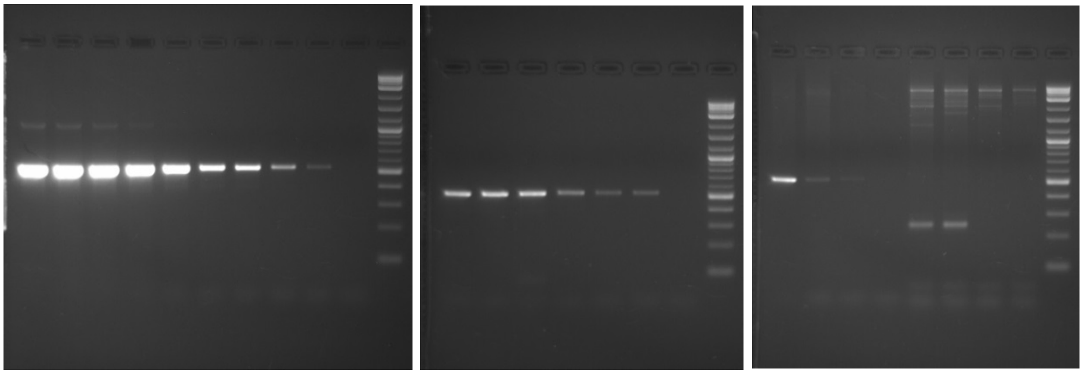

Supplementary Figure 4A.

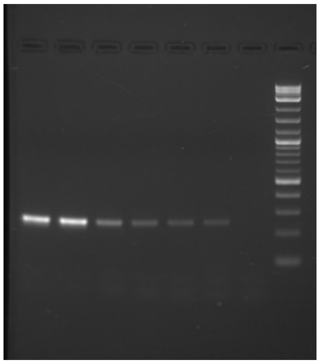

Supplement: Supplementary file 1 — Supplementary information. [file 41598_2020_74474_MOESM1_ESM.pdf]
